# Efficacy of *Bifidobacterium longum* alone or in multi-strain probiotic formulations during early life and beyond

**DOI:** 10.1080/19490976.2023.2186098

**Published:** 2023-03-10

**Authors:** Susan Mills, Bo Yang, Graeme J. Smith, Catherine Stanton, R. Paul Ross

**Affiliations:** a APC Microbiome Ireland, University College Cork, Cork, Ireland; b State Key Laboratory of Food Science and Technology, School of Food Science and Technology, Jiangnan University, Wuxi, Jiangsu, China; c NEOM Food Sector, NEOM, Tabuk, Kingdom of Saudi Arabia; d Food Biosciences Department, Teagasc Food Research Centre, Co Cork, Ireland

**Keywords:** Bifidobacterium, *Bifidobacterium longum* ssp. infantis, *Bifidobacterium longum* ssp. longum, Health, Neonate, Probiotic, Gut microbiota, Necrotizing enterocolitis, Cardiovascular disease, Immunity, Cognitive impairment

## Abstract

The significance of *Bifidobacterium* to human health can be appreciated from its early colonization of the neonatal gut, where *Bifidobacterium longum* represents the most abundant species. While its relative abundance declines with age, it is further reduced in several diseases. Research into the beneficial properties of *B. longum* has unveiled a range of mechanisms, including the production of bioactive molecules, such as short-chain fatty acids, polysaccharides, and serine protease inhibitors. From its intestinal niche, *B. longum* can have far-reaching effects in the body influencing immune responses in the lungs and even skin, as well as influencing brain activity. In this review, we present the biological and clinical impacts of this species on a range of human conditions beginning in neonatal life and beyond. The available scientific evidence reveals a strong rationale for continued research and further clinical trials that investigate the ability of *B. longum* to treat or prevent a range of diseases across the human lifespan.

## Introduction

The human colon is recognized as one of the most densely populated ecosystems on earth with the bacterial component reported to reach 10^14^ cells.^1^ These microorganisms are intricately linked to host physiology and health since they perform several essential functions with consequences not only for the gastrointestinal environment but also for remote organs of the body. Such functions include the development of the host immune system from birth and maintaining immune homeostasis throughout life,^2^ protection from pathogen invasion in the gut via colonization resistance,^3^ energy regulation,^4^ and production of bioactive metabolites and nutrients.^5^ These also influence the many bidirectional interactions between the gut microbiota and other organs/systems of the body including the nervous system,^6^ lungs,^7^ and skin.^8^

Throughout life, several diseases and conditions have been linked with imbalanced gut microbiota profiles. In the preterm neonate, abnormal microbial colonization of the infant intestine is recognized as a risk factor for necrotizing enterocolitis (NEC).^9,10^ Gut microbiome dysbiosis has been associated with gastrointestinal, cardiovascular, metabolic, and neurological diseases, along with autoimmune diseases and allergies across all stages of life.^11,12^

*Bifidobacterium longum* is a commensal gastrointestinal tract (GIT) inhabitant that is recognized as a significant member of the human gut microbiota and is the most abundant species in the infant gut.^13^ It exerts numerous beneficial health effects.^14^ These range from the production of bioactive substances to bifidobacterial surface-associated molecules that interact with the host.^15^ Several *B. longum* strains have thus been developed as probiotics – “live microorganisms which when administered in adequate amounts, confer a health benefit on the host”^16^

The efficacies of this species have been demonstrated in preclinical models and in clinical studies in early human life and beyond. Therefore, in this review, we provide a comprehensive overview of clinical efficacies and biological observations following *B. longum* administration to infants, adults, and elderly in terms of gastrointestinal, cardiovascular, immune, neurological, and respiratory health and disease, as well as host skin based on the results of randomized, double-blind, placebo-controlled trials (RDBPCTs). The relevant literature was obtained following a search in PubMed using the search terms ‘Bifidobacterium longum; double-blind’ with the PubMed filters ‘Randomized Controlled Trial; Clinical Trial.’

Some of the diseases can be categorized as non-communicable diseases (NCDs), linked with genetic, environmental, and lifestyle factors. Yet, research has revealed altered gut microbiota profiles in individuals with NCDs.^17^ The trials have been performed with *B. longum* strains alone or in combination with other strains and/or prebiotics, the latter of which is defined as “a substrate that is selectively utilized by host microorganisms conferring a health benefit.”^18^

In most cases, the strains were administered orally. The results reveal that *B. longum* alone or in combination with other strains and prebiotics can impact various systems in the body and may alleviate disease symptoms or prevent the onset of illness indicating a role for *B. longum* in the prevention and management of several diseases from early life and throughout the human lifespan. However, further clinical trials are warranted before these results can be generalized to appropriate consumers/patients.

## *B. longum* and overview of its beneficial mechanisms of action

*B. longum* is composed to date of four subspecies, *B. longum* ssp. *infantis*, *B. longum* ssp. *longum*, *B*. *longum* ssp. *suis*, and *B*. *longum* ssp. *suillum*. Until recently, the latter two had only been isolated from pigs and calves,^19,20^ however, *B. longum* ssp. *suillum* has since been isolated from the infant gut (unpublished data). Notably, the name *B. longum* ssp. *infantis* is often shortened to *B. infantis* in the literature and *B. longum* ssp. *longum* to *B. longum*. In this review, we refer to the names provided in the literature.

Bifidobacteriales has been identified as the most abundant bacterial class in the infant gut (present at 80.6%) with *B. longum* representing 56.2% of the species.^13^ In breastfed infants, *B. longum* ssp. *infantis* is the most prevalent subspecies^21^ possibly contributed to by its capacity to digest human milk oligosaccharides (HMOs).^22^ In adults, levels of bifidobacteria reduce to 2–14% of relative abundance^23^ and include species such as *B. longum* ssp. *longum*,^24^ *B. adolescentis*, *B. catenulatum*,^25,26^ *B. pseudolongum*, *B. bifidum*, and *B. breve*.^27^ While in the elderly, bifidobacteria levels have been reported to markedly decrease in abundance,^28–30^ and a significant negative correlation has been reported between *B. longum* relative abundance and host age.^31^

*B. longum* is an excellent colonizer of the human gut. For example, following a single oral administration, the strain *B. longum* ssp. *longum* AH1206 persisted in the human gut of 30% of trial subjects for the six-month duration of the study.^32–34^ In contrast, other supplemented strains, such as *Lactobacillus plantarum* (now *Lactiplantibacillus plantarum*), *Lactobacillus acidophilus* La-5 and *Bifidobacterium animalis* ssp. *lactis* BB12 are generally detected in feces in decreasing amounts for a few days after ingestion and rarely beyond 1 week.^35,36^ A longitudinal study investigating the persistence of *B. longum* ssp. *longum* strains in the human gut from infancy revealed that strains confirmed to colonize and persist as early as 90 d after birth were still present at 6 y of age.^37^ Successful colonization of the human gut is partly attributed to the ability of *B. longum* to metabolize host- and diet-derived carbohydrates, such as HMOs and plant polysaccharides that cannot be digested by the host.^15^ Over 13% of the clusters of orthologous gene (COG) families within the pangenome of the *Bifidobacterium* genus are devoted to carbohydrate metabolism.^38^ Among human infant*B. longum* ssp. *longum* strains, Arboleya et al.^32^ identified 22 glycosyl hydrolase families via pan-genome analysis. Interestingly, *B. longum* ssp. *infantis* is specialized for HMO utilization^22^, while *B. longum* ssp. *longum* can also utilize plant-derived polysaccharides, supportive of its ability to colonize both infants and adults.^32–34^

In the gut, *B. longum* metabolizes carbohydrates to short-chain fatty acids (SCFAs), acetate and lactate. In a mouse model, acetate produced in the gut by bifidobacteria has been shown to improve intestinal defenses and protect against infection.^39^ Acetate and lactate reduce the pH in the gut, which is believed to prevent microbiota imbalances and prevent pathogen growth.^40,41^ Indeed, Henrick et al.^42^ reported a trend for increasing fecal pH in breast-fed infants over the past century (from 5.0 to 6.5) that is associated with loss of specialized *Bifidobacterium* species and may pose increased risk for microbiota dysbiosis. In mice, acetate has been shown to promote intestinal antibody immunoglobulin (Ig)A responses in the gut via the G-protein coupled receptor GPR43.^43^ Acetate can be used by butyrate-producing bacteria in the gut, such as *Faecalibacterium prausnitzii*, to produce butyrate.^44^ Butyrate is used as an energy source by gut epithelial cells and is involved in several physiological functions including intestinal barrier function,^45^ immunity,^46^ and brain function.^47^ Lactate can also cross the blood–brain barrier and behave as a neuromodulator in the brain.^48^ Another metabolite produced by *B. longum* ssp. *infantis* following growth on HMOs is indole-3-lactic acid, a tryptophan metabolite that has been shown to significantly decrease inflammation in gut epithelial cells.^49^ Indole-3-lactic acid was identified as the anti-inflammatory molecule in *B. infantis* secretions that prevents transcription of the inflammatory cytokine interleukin (IL)-8 in immature, but not mature, intestinal enterocytes.^50^ Indeed, indole-3-lactic acid exerts different anti-inflammatory, anti-viral, and cell development effects on immature and mature enterocytes and has been proposed as a potential therapeutic in the prevention and treatment of NEC in premature infants.^51^

Many bifidobacteria produce polysaccharides including capsular polysaccharides (CPSs) that are bound to the cell surface and exopolysaccharides (EPSs) that are loosely attached to the bacterial cell or secreted into the surrounding environment.^52^ These molecules serve to protect bacterial cells against harsh environments encountered in the gastrointestinal tract but can also be involved in crosstalk with the gut environment. Bifidobacterial EPSs have been shown to enhance adhesion to eukaryotic cell lines and depending on chemical and physical properties have been shown to elicit or reduce an immune response.^53^ Surface-associated EPSs have been shown to reduce pathogen colonization in mice.^54^ More recently, Yan et al.^55^ reported that a ropy-EPS-producing *B. longum* ssp. *longum* strain alleviated the symptoms of dextran sodium sulfate- (DSS-)induced colitis in mice and reduced inflammation by decreasing pro-inflammatory cytokines. However, a non-ropy-EPS-producing *B. longum* ssp. *longum* strain failed to decrease pro-inflammatory cytokine levels. Furthermore, the ropy EPS-producing strain maintained the expression of genes involved in mucosal barrier function after DSS challenge but a non-EPS producing strain failed to maintain such gene expression.

Certain *Bifidobacterium* species including *B. longum* have been shown to produce serine protease inhibitors (serpins) and harbor serpin-encoding genes.^56,57^ Serpins serve to promote bifidobacterial colonization as they protect bacterial cells from host-derived proteases. Indeed, Ivanov et al.^56^ reported that serpin from *B. longum* effectively inhibited eukaryotic elastase-like proteases, leading them to speculate on its role in immunomodulation given that elastase is released at sites of intestinal inflammation by activated neutrophils. The serpin-producing *B. longum* strain NCC 2705 was capable of attenuating gliadin-induced immunopathology in a mouse model of gluten sensitivity, while its serpin-knockout counterpart failed to elicit such an effect.^58^ Most recently, the concept of the ‘gut serpinome’ has been introduced – serpins produced by the gut microbiota – given the capacities of these serpins to inhibit proteases involved in the pathogenesis of inflammatory bowel disease (IBD) and their potential for innovative therapies.^59^

Interestingly, not all *B. longum* strains can alleviate disease symptoms as already noted.^55^ Chen et al.^60^ investigated the impact of three conjugated linoleic acid- (CLA)-producing *B. longum* strains on DSS-induced colitis in mice. Only one strain, *B. longum* CCFM681, proved capable of alleviating colitis by inhibiting pro-inflammatory pathways, protecting the intestinal mechanical barrier, and modulating the gut microbiota. These beneficial effects are correlated with CLA production and while all three strains were confirmed CLA producers *in vitro*, *B. longum* CCFM681 produced significantly more CLA in the colon than the other two strains, suggesting that CLA production, in this case, was responsible for relieving colitis. Another study reported that *B. longum* strains with different genotypes in the arginine biosynthesis pathway had different abilities for protecting a d-galactose-induced aging mouse model against host aging – proposed to be associated with their differing abilities to alter the gut microbiota metabolome.^31^

### *B. longum* effects in infants and children

#### Gastrointestinal health and disease

##### Necrotizing enterocolitis and late-onset sepsis

Preterm infants are at increased risk of developing the intestinal inflammatory disease, NEC, due to the underdeveloped gastrointestinal environment and preterm microbiome signatures.^61,62^ NEC is estimated to affect 5–12% of preterm infants (<1500 g at birth) with mortality rates as high as 20–30%.^61,63^ Several prenatal, perinatal, and neonatal risk factors have been identified,^63^ including intestinal immaturity and abnormal microbial colonization of the infant intestine.^9,10^ Prior to the onset of NEC in preterm infants, the intestinal microbiome has been characterized by reduced microbial diversity,^61,64,65^ increased relative abundance of Proteobacteria and decreased relative abundances of Firmicutes and Bacteroidetes,^66^ including lower levels of commensals such as bifidobacteria.^67^ A recent study using mice revealed that NEC microbiota (from patients with NEC) causes intestinal injury in germ-free mice following fecal microbiota transplantation.^68^ Thus, the use of beneficial bacterial strains to prevent and treat NEC is an area of continued and growing interest.^61,69–71^

In a prospective multicentre RDBPCT (*ProPrems* trial), Jacobs et al.^72^ investigated the impact of a combination of bacterial strains on the occurrence of late-onset sepsis in preterm infants (born before 32 weeks’ gestation). The formulation included *B. longum* ssp. *infantis* BB02, *Streptococcus thermophilus* TH-4, and *B. animalis* ssp. *lactis* BB-12 (in maltodextrin powder) and the placebo group received maltodextrin. Bacterial strains were associated with a 54% reduction in NEC of Bell stage 2 or more in very preterm infants; however, they did not reduce definite late-onset sepsis or mortality. A follow-on study revealed that the bacterial formulation was associated with increased *Bifidobacterium* in the gut microbiota of the very preterm infants (*p* < 0.001) and decreased *Enterococcus* levels (*p* = 0.02), suggesting that *Bifidobacterium* may have a protective effect against NEC.^73^ The bacterial formulations of *B. longum, L. acidophilus*, *Lactobacillus rhamnosus* (now *Lacticaseibacillus rhamnosus*), and *Saccharomyces boulardii* showed a trend toward lowering NEC (4% versus 12%) in very low birth weight neonates in a randomized, double-blinded controlled trial where breast milk served as the control (no placebo included).^74^ However, the authors suggest that the use of breast milk in the control group may have narrowed the differences between the two groups given the beneficial properties associated with breast milk. Cross-contamination between the two groups in the hospital setting could also have impacted the results where the control group may have acquired strains from the formulation, although this was not assessed in the study. Furthermore, 73% of infants in the control group were born by cesarean delivery versus 52% in the test group; however, cesarean-delivered infants may have benefited more from the intervention given that colonization of these infants with beneficial microbes, such as *Bifidobacterium* is delayed.^75^

#### Gastroschisis

Gastroschisis describes a ventral body wall defect where the bowel exits the infant’s body *in utero*.^76^ Infants born with this condition undergo long periods of gastric suctioning and hospital stays. In a randomized, placebo-controlled, blinded pilot study, the administration of *B. longum* ssp. *infantis* to infants with gastroschisis partially attenuated the significant gut dysbiosis observed in these infants; however, there was no impact on the length of hospital stay.^77^ Specifically, the gut microbiota of infants born with gastroschisis was dominated by *Enterobacteriaceae*, *Staphylococcaceae*, *Streptococcaceae*, and *Enterococcaceae*. Long-term studies of infants born with this condition are limited but increased prevalence of obesity and hypercholesterolemia in later childhood and teen years have been documented in this group,^78^ which could be linked to the early gut microbiota dysbiosis.^79^ *B. longum* ssp. *infantis* exposure was associated with colonization with moderate numbers of *Bifidobacteriaceae* but the effect was most pronounced after gastric suction had ended and the strain was fed orally (as opposed to the twice-daily 1-h exposure of the gastric mucosa to the strain). The authors suggest that more pilot studies with more frequent and/or higher doses of strains are needed to decipher whether administering a bacterial formulation during gastric suctioning has any impact. Future studies would also benefit from a larger sample size (given that only 24 infants were enrolled in the study) and better coordination in the timing of sample collection and number of samples collected per infant. However, the results of this pilot trial suggest that *B. longum* ssp. *infantis* could have a role to play in the therapy of gastroschisis. Further studies are warranted that address the most appropriate strains for infants with gastroschisis, the precise method of treatment, dosage, and frequency, and the long-term benefits of such for health and disease evasion.

#### Childhood diarrhea

In developing countries, childhood diarrhea is the second leading cause of infant mortality (respiratory diseases being the first)^80^ and rotavirus is the leading cause of acute diarrhea-related deaths worldwide in children under the age of five.^81^ The strain *B. longum* ssp. *infantis* CECT7210 (*B. infantis* IM1) isolated from the feces of a breast-fed infant has been shown to inhibit rotavirus infection of cell lines and provide preliminary protection against virus infection in a mouse model.^82^ Using this strain in supplemented infant formula, Escribano et al.^83^ investigated its effectiveness in reducing diarrhea incidence in healthy term infants during 12 weeks of intervention in a multicentre RDBPCT. In the overall study period, the median diarrhea events per infant were recorded as 0.29 ± 1.07 for the control group and 0.05 ± 0.28 for the *B. infantis* IM1 group (*p* = 0.059), and this reached significance by week 8 (*p* = 0.047). However, it should be pointed out that the incidence of diarrhea among the whole sample was small overall, which could be due to the young age of the participants (<3 months) who would harbor protective maternal antibodies. The strains *B. longum* BORI and *L. acidophilus* AD031 were associated with a significant reduction in diarrhea duration (by 1.2 d) in infants hospitalized with rotavirus infection (*p* = 0.001) in a RDBPCT that lasted for 3 d, while fever duration, diarrhea, and vomiting frequencies tended to be reduced by the strains.^84^ The short duration of the trial suggests that a longer treatment period could result in better outcomes for the parameters tested.

#### Irritable bowel syndrome

Formulations containing *B. longum* have also generated promising results in improving symptoms of irritable bowel syndrome (IBS) and ulcerative colitis (UC) in children. IBS symptoms include abdominal pain and alterations in bowel habits, but the exact pathogenesis is unclear.^85^ A multicentre, crossover RDBPCT reported that administration of a mixture of *B. infantis M*-63, *B. breve M*-16 V, and *B. longum* BB536 for 6 weeks to children with IBS resulted in a complete resolution of abdominal pain in a significantly higher number of children compared with placebo (*p* = 0.006), and significantly improved abdominal pain frequency (*p* = 0.02).^86^ Moreover, 48% of children with IBS reported an improvement in quality-of-life following treatment versus 17% in the placebo group (*p* = 0.001). However, it is not known if the washout period of 2 weeks was sufficient to prevent a “carryover” effect between treatments, which can be a limitation of crossover trials.

#### Inflammatory bowel disease

UC describes a recurring inflammation of the colon and rectum with symptoms of abdominal pain, bloody diarrhea, fecal urgency, and tenesmus.^87^ Along with Crohn’s disease (CD) – an inflammatory disease that can affect any part of the intestine, it is classified as an IBD. In a 1-y-long, RDBPCT, consumption of the probiotic blend VSL#3® (consisting of four strains of *Lactobacillus*, three strains of *Bifidobacterium* including *B. longum* and *B. infantis*, and one strain of *Streptococcus salivarius* subsp. *thermophilus*, see Table 1 for details) by children with newly diagnosed UC in conjunction with IBD therapy demonstrated significant efficacy for inducing and maintaining remission (*p* < 0.001) compared with placebo and IBD therapy.^88^ A significantly lower rate of relapse was recorded in the VSL#3® group (*p* = 0.014). In this case, the authors concluded that the high bacterial counts of 3 × 10^11^ cells/g contributed to the success of the formulation along with the large number of different strains. However, the small sample size (*n* = 29) used in this trial suggests that confirmatory trials should be conducted with higher patient numbers.

**Table 1.** An overview of clinical trials investigating the impact of *B. longum* on infants and children.

Condition/Disease/Biological Parameter	Participants; Age	Formulation	CFU; Dose; Duration	Clinical Effects and Biological Observations of Intervention Group Compared with Placebo Group	Reference; Trial ID
*Gastrointestinal Conditions*					
Late onset sepsis	1099; Very preterm infants (<1500 g)	*B. longum* ssp. *infantis* BB02, *S. thermophilus* TH-4, *B. animalis* ssp. *lactis* BB-12	10^9;^ 2 daily doses until hospital discharge or term corrected age	Significant reduction in NEC of Bell stage 2 or more; No reduction in definite late-onset sepsis or all-cause mortality	Jacobs et al.^72^ ACTRN012607000144415 (Multicentre)
Time to reach full enteral feeds in very low birth-weight newborns	104; Very Low Birth Weight (75–1499 g)	*B. longum*, *L. acidophilus*, *L. rhamnosus*, *S. boulardii*	1.25 x 10^9;^ 1 daily dose, From initiation of enteral feeds till hospital discharge	Trend towards reduced NEC; No impact on feed tolerance	Shashidhar et al.^74^ CTRI/2012/08/002853
Gastroschisis	24; Gestational age at birth > 34 weeks	*B. longum* ssp. *infantis* ATCC 15,697	10^9;^ 2 daily doses for 6 weeks or until hospital discharge	Higher *Bifidobacteriaceae*, lower *Clostridiaceae*; Trend towards lower *Enterobacteriaceae*, *Enterococcaceae*, *Staphylococcaceae*, & *Streptococcaceae*; No impact on length of hospital stay	Powell et al.^77^ NCT01316510
Diarrhea	151; Term infants (< 3 months)	*B. longum* ssp. *infantis* CECT7210	10^7;^ daily, 12 weeks	Significantly reduced diarrhoea episodes at week 8	Escribano et al.^83^ NCT02096302 (Multicentre)
Rotavirus disease	57; 9–16 months	*B. longum* BORI, *L. acidophilus* AD031	2 x 10^10^ *B. longum*, 2 x 10^9^ *L. acidophilus*; 2 daily doses, 3 d	Significantly reduced duration of diarrhoea (by 1.2 d); Tended to ameliorate duration of fever, frequencies of diarrhoea & vomiting	Park et al.^84^
Irritable bowel syndrome	48; 8–17.9 y	*B. infantis M*-63, *B. longum* BB536, *B. breve M*-16V	3 x 10^9^ *B. longum*, 1 x 10^9^ *B. infantis*,1 x 10^9^ *B. breve*; 6 weeks	Resolution of abdominal pain in significantly higher number of children; Significantly improved frequency of abdominal pain; Improvement in Quality of Life significantly higher	Giannetti et al.^86^ NCT02566876 (Multicentre)
Ulcerative colitis	29; 1.7–16.1 y	VSL#3: *B. longum*, *B. breve*, *B. infantis*, *L. paracasei*, *L. plantarum*, *L. acidophilus*, *L. delbrueckii* subsp. *bulgaricus*, *S. salivarius* subsp. *thermophilus*	4.5 x 10^11−^1.8 x 10^12^ (age-dependent); 1 year	Significant efficacy for inducing & maintaining remission	Miele et al.^88^
***Immunity***					
Immunity & Gut microbiota composition	264; healthy term newborns	*B. longum* BB536	1 x 10^7;^ 12 months	Significantly elevated levels of IF-γ secretion cells; Ratio of IF-γ/IL-4 secretion cells significantly higher; Faecal bifidobacteria counts & bifidobacteria/*Enterobacteriaceae* ratio significantly higher	Wu et al.^89^
Severe sepsis & levels of pro- & anti-inflammatory cytokines	100; 3 months to 12 y	VSL#3: *B. longum*, *B. breve*, *B. infantis*, *L. paracasei*, *L. plantarum*, *L. acidophilus*, *L. delbrueckii* subsp. *bulgaricus*, *S. salivarius* subsp. *thermophilus*	4.5 x 10^11;^ 7 d	Significant decrease in pro-inflammatory cytokines; Significant increase in anti-inflammatory cytokines; Significant reduction in Sequential Organ Failure Assessment score; Lower incidence of healthcare-associated infections; Reduced duration of stay in intensive care unit	Angurana et al.^90^
***Cardiovascular Health***					
Dyslipidaemia	38; 10.8 ± 2.1 y	*B. longum* ssp. *longum* BL04, *B. animalis* ssp. *lactis* MB 2409, *B. bifidum* MB 109B	3 x 10^9^, daily; 3 months	Significantly reduced total cholesterol & LDL-cholesterol	Guardamagna et al.^91^
***Developmental Disorders***					
Autism spectrum disorder	26; 4–5 y	*B. infantis* Bi-26, *L. rhamnosus* HN001, *B. lactis* BL-04, *L. paracassei* LPC-37, & FOS	10^10^ daily; 108 d	Significantly increased beneficial bacteria when compared with baseline; Significantly reduced levels of suspected pathogens; Significantly increased SCFA & homovanillic acid; Significantly reduced serotonin; Improved gastrointestinal autism severity	Wang et al.^92^
***Respiratory Health and Seasonal Allergies***					
Common winter diseases	135; 3–7 y	*B. infantis* R0033, *B. bifidum* R0071, *L. helveticus* R0052, FOS	3 x 10^9^ 750 mg; once daily; 3 months	Reduced the number of children who suffered at least one winter disease by 25% & limited the number of school days lost	Cazzola et al.^93^
Upper respiratory illnesses	219; 2–6 y	*B. longum* BB536	5 x 10^9;^ 5 d/week for 10 months	Significantly reduced duration of sore throat; Numerically reduced duration of runny nose & cough; Increased *Faecalibacterium* in gut microbiota	Lau et al.^94^ NCT02434042
Seasonal allergic rhinitis & intermittent asthma	40; 9 ± 2.2 y	*B. longum* BB536, *B.* *infantis* M-63, *B.* *breve* M-16V	3 x 10^9^ 1 x 10^9^ 1 x 10^9;^ once daily; 8 weeks	Significantly relieved nasal symptoms of allergic rhinitis & improved quality of life	Miraglia Del Giudice et al.^95^ NCT02807064.
***Skin Health***					
Eczema development in at-risk infants	241 pregnant women. Supplementation began 2 months before delivery and during first 2 months of breast feeding. 205 infants assessed	*B. longum* BL999, *L. rhamnosus* LPR or *B. longum* BL999, *L. paracasei* ST11	1 x 10^9^ daily; 4 months	Both formulations significantly reduced the risk of developing eczema in at-risk infants	Rautava et al.^96^
Eczema development in at-risk infants	245 Infants; newborn	*B. longum* BL999, *L. rhamnosus* LPR	~9 x 10^7^ ~ 2 x 10^8;^ daily; 6 months	No impact	Soh et al.^97^
Moderate atopic dermatitis	50; 4–17 y	*B. longum* CECT 7347, *B. lactis* CECT 8145, *L. casei* CECT 9104	1 x 10^9^ daily; 12 weeks	Significantly reduced SCORAD & reduced eczema spread & intensity	Navarro-López et al.^98^ NCT02585986

### Immunity

Probiotics have been defined as immunostimulatory – for example, they act against infection by inducing production of the pro-inflammatory cytokine IL-12 that activates natural killer (NK) cells and develops T helper (Th)1 cells; or immunoregulatory – they promote production of the anti-inflammatory cytokine IL-10 and T regulatory cells (T-regs).^99^ Bifidobacteria have been shown to modulate specific immune cells and pathways in both animals and humans. The mechanisms involved are not yet fully understood and vary from strain to strain but they can induce pro- or anti-inflammatory effects. For the adaptive immune system, the balance of T-cell subsets Th1, Th2, Th17, and T regulatory cells [Tregs]) is critical to homeostasis.^100,101^

During pregnancy, a bias toward Th2 cells protects the fetus.^102^ Th2-type cytokines tend to produce an anti-inflammatory response.^103^ After birth, the development of the Th1 immune response (Th1-type cytokines tend to be pro-inflammatory)^103^ can reset the Th1/Th2 balance, and it is suggested that exposure to environmental microbes plays a critical role in this.^89^ In infants, high levels of circulating Th2-associated chemokines and low levels of Th1-chemokines have been associated with allergic disease and sensitization.^104^ Wu et al.^89^ investigated the impact of administering *B. longum* BB536 to healthy newborn infants on the immune response and intestinal microbiota over a 12-month period where interferon-γ (IF-γ) secretion cells were used to represent Th1 cytokines, and IL-4 secretion cells were used to represent Th2 cytokines. At 7 months of age, infants in the BB536 group had significantly elevated levels of IF-γ secretion cells compared with the control group (*p* = 0.007), and the ratio of IF-γ/IL-4 secretion cells was significantly higher in the supplemented group (*p* = 0.044). By 2 and 4 months of age, the fecal bifidobacteria counts and the bifidobacteria/*Enterobacteriaceae* ratio were significantly higher in the BB536 group (*p* < 0.05). However, *B. longum* BB536 had no impact on serum antibody titers following vaccination with vaccines for hepatitis B, poliomyelitis, and Diphtheria tetanus toxoid and pertussis when compared with the control group. It is possible that the healthy term infants in this RDBPCT already had adequate antibody responses to the vaccines, thus BB536 did not exert a further effect. This has been reported in other studies following the administration of beneficial bacteria.^105,106^

In children with severe sepsis, 7 d of supplementation with VSL#3® was associated with significant reductions in the pro-inflammatory cytokines IL-6 (*p* = 0.001), IL-12p70 (*p* = 0.001), IL-17 (*p* = 0.01), and tumor necrosis factor-α (TNF- α) (*p* = 0.01) compared with the placebo group.^90^ The anti-inflammatory cytokine IL-10 and transforming growth factor-β1 were significantly increased (*p* = 0.02 and *p* = 0.01, respectively). However, caution should be exerted when interpreting these results since cytokine profiling was not performed in duplicate. The Sequential Organ Failure Assessment score was significantly lower in the VSL#3® group compared with placebo on day 7 (1 versus 3). Duration of intensive care unit (ICU) stay was also reduced in the VSL#3® group compared with placebo (6.5 d versus 9). There was also a non-significant trend toward a lower incidence of healthcare-associated infections in the VSL#3® group compared with placebo (14% v 20%). Despite these promising findings, caution is warranted in their interpretation given that the percentage of patients with septic shock in the placebo was greater than that in the test group (60% versus 48%, respectively) which could have influenced these results. Thus, further trials with better randomization are required to confirm these findings. For further details of these trials, see Table 1.

### Cardiovascular health

Cardiovascular diseases (CVDs) affect the heart and blood vessels and include a range of complications and conditions from abnormal heart rhythms to heart failure, heart attack, and stroke, as examples. The World Health Organization (WHO) estimates that CVDs are responsible for approximately 17.9 million deaths annually with more than 4 out of 5 deaths due to heart attacks and strokes.^107^ High blood pressure and high cholesterol are risk factors for CVDs. Statins are generally prescribed to lower low-density lipoprotein (LDL) cholesterol and thus reduce cardiovascular events and mortality.^108^ However, some patients report side effects from statin therapy such as muscle pain, and while a recent systematic review reported that only a small minority of symptoms are due to statins, the development of new-onset diabetes mellitus was significantly higher when taking statins.^109^ Thus, there is a need for alternative treatments that lower cholesterol without subsequent side effects.

#### Blood lipid profiles

Lipoprotein disorders can be inherited and can lead to the early development of atherosclerosis in children,^110^ which can manifest as CVDs in adulthood.^111^ International guidelines recommend good nutrition as the primary approach to reducing excess cholesterol in children, particularly LDL cholesterol, while the use of drug treatment is a last-resort option when dietary treatment and recommended supplements (e.g., plant sterols) prove insufficient.^112^ Thus, cholesterol-lowering bacterial strains could offer a viable strategy, in conjunction with healthy nutrition, to control cholesterol levels in children. Guardamagna et al.^91^ investigated the impact of a three-strain formulation on lipid profiles in children (10.8 ± 2.1 y) affected by primary dyslipidemia. Enrolled children had to have serum total cholesterol levels greater than their age- and sex-specific 90^th^ percentile.^113^ Exclusion criteria included secondary dyslipidemia, obesity, or overweight, disorders of the renal or endocrine systems or liver and chronic diseases that required treatment. The formulation consisted of three different *Bifidobacterium* species, namely *B. longum* ssp. *longum* BL04, *B. animalis* ssp. *lactis* MB 2409, and *B. bifidum* MB 109B. The mixture was capable of cholesterol assimilation, bile salt hydrolase activity, and conversion of linoleic acid to CLA. Assimilation refers to the ability of bacteria to assimilate cholesterol into the bacterial cell membrane, thus reducing cholesterol reabsorption in the gut.^114^ Bacterial bile salt hydrolase deconjugates bile salts into bile acids that are then excreted from the body in the feces.^115^ CLA has demonstrated a host of beneficial activities in animal studies and human cell lines, including protection against obesity and atherosclerosis.^116^ In the RDBPC crossover study, 3 months of treatment significantly reduced total cholesterol by 3.4% and LDL-cholesterol by 3.8% compared with placebo (*p* = 0.001).^91^ Despite this, LDL and total cholesterol values of the participants remained above the acceptable values of <110 mg/dl and<170 mg/dl, respectively, for children^117^ following treatment (at 135 and 212 mg/dl, respectively), bringing into question the physiological relevance of the results and whether the duration of the trial was adequate. Furthermore, all participants in the study were given a dietary regimen (STEP 1 diet) by a trained dietitian 4 weeks prior to commencement of the trial, which itself resulted in statistically significant reductions in total and LDL cholesterol in the placebo group compared with baseline values. Indeed, while the bacterial formulation resulted in 4.6% (*p* = 0.0001) and 8.2% (*p* = 0.0001) reductions in total and LDL cholesterol from the baseline, respectively, the placebo resulted in reductions of 3.5% (*p* = 0.001) and 6.3% (*p* = 0.0007%), respectively. The crossover nature of the study could be a contributing factor if the 4-week washout period was too short to prevent potential carryover effects in the formulation. Thus, further studies are warranted in animals and humans to decipher the bacterial formulations, duration, and dosage regimens that generate physiologically meaningful reductions in LDL and total cholesterol. For further details of these trials, see Table 1.

#### Developmental disorders

The microbiota-gut-brain axis describes the bi-directional communication pathways between the gut microbiota and its metabolites, the central, enteric, and autonomic nervous systems, and the hypothalamic-pituitary-adrenal axis.^118^ Certain members of the gut microbiota, including *B. longum*, have been shown to produce neurochemicals such as the major inhibitory neurotransmitter gamma amino butyric acid,^119^ or are involved in the regulation of host serotonin biosynthesis.^120,121^ Furthermore, bacterially produced SCFAs are involved in the microbiota-gut-brain axis with the potential to influence mood, cognition, and brain disorder etiology, directly or indirectly.^122^

#### Autism spectrum disorders

In 2012, it was estimated that 1 in 160 children globally had a pervasive developmental disorder including an autism spectrum disorder (ASD).^123^ But the reported prevalence has since increased, and in Ireland alone, the prevalence rate for ASD in children was estimated at 1.5%.^124^ Comorbidities include gastrointestinal issues such as abdominal pain, constipation, and diarrhea.^125^ Wang et al.^92^ performed a RDBPCT in children with ASD to investigate the impact of a synbiotic formulation on ASD symptoms as well as gut microbiota composition, SCFA concentrations, and levels of neurotransmitters. A synbiotic is defined as “a mixture comprising live microorganisms and substrate(s) selectively utilized by host microorganisms that confers a health benefit on the host.”^126^ In this case, the synbiotic consisted of four bacterial strains with the prebiotic fructo-oligosaccharides (FOS). However, before the intervention took place, differences in fecal microbiota composition, SCFA production, and plasma neurotransmitters were investigated between children diagnosed with ASD and normal children. Interestingly, children with ASD had significantly lower levels of beneficial bacteria, *B. longum* and Bifidobacteriales in terms of relative abundance (*p* < 0.05) and significantly higher levels of *Ruminococcus* and *Clostridium* (*p* < 0.01). Levels of the SCFAs butyrate, propionate, and acetic acid were also significantly lower in children with ASD (*p* < 0.05). Furthermore, these children were found to be in a hyper-serotonergic state and had significantly decreased levels of homovanillic acid (*p* < 0.001), an indicator of dopaminergic activity in the central nervous system.^127^ The synbiotics, which consisted of *B. infantis* Bi-26, *L. rhamnosus* HN001, *B. lactis* BL-04, *Lactobacillus paracasei* (now *Lacticaseibacillus paracasei*) LPC-37, and the prebiotic FOS, was administered for 108 d in total, and the analysis of parameters was performed at 30, 60 and 108 d. The synbiotic was associated with significantly increased beneficial bacteria when compared with baseline (day 0) including *B. longum* (days 30 and 60, *p* < 0.05; day 108 *p* < 0.001) and reduced levels of suspected pathogens such as *Clostridium* (day 108, *p* < 0.05) compared with the placebo group (children with ASD receiving placebo). The synbiotic also resulted in significant elevations in individual SCFA levels, significantly increased homovanillic acid, and significantly reduced serotonin, which were not observed in the placebo group. However, as the authors point out, the synbiotic failed to modulate a number of neurotransmitters and metabolites including glutamine, glutamic acid, acetylcholine, gamma amino butyric acid, arginine, histidine, and histamine. But the synbiotic did improve gastrointestinal symptoms in participants and reduced autism severity as assessed by the Autism Treatment Evaluation Checklist (ATEC). Further studies are warranted with larger sample sizes and to determine if FOS is also necessary for the observed effects.

#### Respiratory health and seasonal allergies

Upper respiratory tract infections describe viral or bacterial infections of the nose, pharynx, larynx, sinuses, and large airways, and have been identified as one of the top three diagnoses in outpatient settings.^128^ In 2003, it was estimated that non-influenza-related, viral respiratory tract infections in the United States posed an annual economic burden above $22 billion.^129^ Seasonal allergies, including hay fever or allergic rhinitis, occur at a certain time of the year when pollen counts are high with symptoms of runny nose, watery eyes, coughing, and sneezing. In allergic 2-year-old children, the gut microbiota has been characterized by lower bifidobacteria, lactobacilli, and *Bacteroides* and higher levels of aerobic microorganisms including *Staphylococcus aureus* compared with non-allergic children. Thus, interventions with beneficial bacteria have the potential to prevent or reduce the severity of respiratory illnesses and seasonal allergies through modulation of the gut microbiota, its functionality, and host immunity.

In a multicentre RDBPC pilot study, children who had previously suffered at least three episodes of common winter diseases including ear, nose, and throat, respiratory tract, or gastrointestinal illnesses were administered a synbiotic formulation for 3 months to determine its efficacy at preventing common winter diseases.^93^ The synbiotic consisted of *B. infantis* R0033, *B. bifidum* R0071, *L. helveticus* R0052, and FOS. Compared with placebo, the synbiotic resulted in a 25% relative risk reduction in the percentage of children who suffered at least one winter disease during the treatment period (*p* = 0.045) and limited the number of school days lost (*p* = 0.043). However, a potential limitation of the study is the unbalanced number of children in each group (*n* = 73, placebo; *n* = 62, test) due to enrollment difficulties that could have resulted in a study bias. Despite this, the results are promising and should help in the strategic design of a larger clinical trial.

Consuming *B. longum* BB536 for 10 months alleviated the symptoms of upper respiratory illnesses in children aged 2–6 y old in a parallel RDBPCT.^94^ Specifically, the strain was associated with a reduced duration of sore throat by 46% (*p* = 0.018), runny nose by 15% (*p* = 0.087), and cough by 16% (*p* = 0.087) compared with the placebo. Interestingly, the analysis of the gut microbiota revealed an increase in the genus *Faecalibacterium* in the BB536 group between 0 and 10 months, which was not observed in the placebo group (*p* < 0.05). In the previous section on Immunity, the same strain was shown to increase IF-γ secretion cells in healthy-term newborns.^89^ The same strain combined with *B.* *infantis* M-63 and *B.* *breve* M-16 V proved effective for significantly relieving nasal symptoms of allergic rhinitis (nasal itching, nasal obstruction, sneezing, rhinorrhea, and itchy eyes; *p* < 0.005) and improving quality of life (*p* < 0.001) in children suffering from seasonal allergic rhinitis and intermittent asthma due to pollen.^95^ However, the small sample size used in this trial (n = 40) suggests that further trials with larger participant numbers are needed to confirm the findings.

#### Skin health

While the large intestine is estimated to carry 10^14^ bacterial cells, the skin microbiome is said to harbor 10^11^.^1^ Common skin disorders have been associated with imbalances in the skin microbiome such as acne vulgaris, which has been associated with *Cutibacterium acnes* type 1A^130,131^ and atopic dermatitis, associated with increased *S. aureus* abundance^132,133^ as examples. For extensive reviews on this topic, the reader is referred to De Pessemier et al.^8^ and O’ Sullivan et al.^134^.

The gut – skin axis describes the bidirectional communication between the gut ecosystem and skin and is generally mediated via the host’s immune system.^8^ Indeed, many skin disorders have been associated with altered gut microbiota (reviewed extensively by De Pessemier et al.^8^. For example, atopic dermatitis has been associated with reduced gut levels of Bacteroidetes, *Akkermansia*, and *Bifidobacterium*, and higher levels of *F. prausnitzii*, *Clostridium*, and *Escherichia coli*.^135–141^ Following a review of the evidence, the World Allergy Organization recommended probiotic supplementation for prevention of allergy in infants, albeit the evidence was described as ‘very low quality’.^142^

A small number of RDBPCTs have been performed with *B. longum*-containing formulations, particularly in infants and children, with promising results.

##### Atopic dermatitis

The inflammatory skin disorder, atopic dermatitis, is said to be the most common inflammatory skin disease.^143^ With symptoms of dry, itchy, cracked, and sore skin, atopic dermatitis can significantly impact quality of life. Paller et al.^144^ reported that atopic dermatitis also puts patients at risk of other non-allergic conditions, such as anxiety, attention deficit hyperactivity disorder (ADHD), and depression and it is also associated with bacterial and viral cutaneous and extra-cutaneous infections. While moisturizers are considered standard therapy, a recent review of over-the-counter therapies revealed that not all are beneficial with some being deleterious.^143^ Thus, microbial interventions that can modulate the inflammatory status of the skin pose a highly attractive option.

Rautava et al.^96^ investigated the impact of maternal supplementation with different bacterial strains during the last 2 months of pregnancy and the first 2 months of breastfeeding on reducing the risk of eczema development in high-risk infants in a parallel RDBPCT. Infants were considered high risk if the mother presented with atopic sensitization and had a history of or active allergic disease. Two formulations were assessed: *B. longum* BL999 with *L. rhamnosus* LPR and BL999 with *L. paracasei* ST11, and the infants were assessed for 24 months. Both formulations were deemed safe and significantly reduced the risk of developing eczema (*p* < 0.001 for both). More specifically, while 71% of infants in the placebo group developed eczema, only 29% in each intervention group were recorded as having eczema. Chronically persistent eczema was reported in 26% of the placebo group but only in 10% of the BL999 + LPR group (*p* = 0.016) and 6% of the BL999 + ST11 group (*p* = 0.003). The bacterial strains had no impact on the risk of atopic sensitization in infants. Interestingly, supplementing at-risk infants with *B. longum* BL999 and *L. rhamnosus* LPR in commercially available cow’s milk formula for the first 6 months of life had no impact on eczema incidence or atopic sensitization during the first year of life.^97^ Rautava et al.^96^ suggest that prenatal probiotic supplementation may alter the maternal intestinal and vaginal microbiota, which provide important colonizing inocula to the newborn infant.^62^ The authors also suggested that maternal administration of bacterial strains may alter the immuno-physiology of the foetoplacental unit. Indeed, the same research group reported such an observation in humans following a RDBPCT whereby strains of *B. lactis* or *B. lactis* with *L. rhamnosus* GG significantly altered toll-like receptor (TLR)-related gene expression in both the placenta and fetal gut.^145^ Furthermore, evidence suggests that maternal intestinal microbes can be transferred to breast milk via the enteromammary pathway^146^ and orally ingested probiotic strains have been identified in mother’s breast milk,^147,148^ which could provide a means of transferring the strains directly to the breastfed infant. Indeed, for a single breastfeeding mother-infant pair of cesarean delivery, Kordy et al.^149^ identified a distinct *B. breve* strain in the infant stool, maternal breast milk, and maternal rectum suggesting transfer of maternal gut bacteria to the mammary gland and then to the infant. Furthermore, beneficial bacteria in breast milk may be supported by HMOs.^150^

In a group of young participants (aged 4–17 y), 12 weeks of supplementation with the formulation *B. longum* CECT 7347, *B. lactis* CECT 8145, and *L. casei* CECT 9104 reduced the SCORAD (Scoring Atopic Dermatitis) index during the supplementation period, and reduced eczema spread and intensity.^98^ After 12 weeks, the mean reduction in the SCORAD index for the intervention group was 19.2 points greater than in the placebo group (*p* < 0.001). Furthermore, the proportion of days of topical steroid use was also significantly less in the test group (*p* < 0.003). However, the dose of topical corticosteroid treatment was not recorded. For further details of these trials, see Table 1.

## *B. longum* effects in adults

### Gastrointestinal health and disease

#### Inflammatory bowel disease

UC has a prevalence of 156 to 291 cases per 100,000 persons per year and is more prevalent in adults than children.^151^ A synbiotic that consisted of *B. longum* 536 isolated from healthy rectal epithelium combined with a FOS-inulin prebiotic (Synergy 1; Orafti, Tienen, Belgium) was associated with a significant reduction in mucosal inflammatory markers, improved appearance of chronic inflammation and regeneration of epithelial tissue following 4 weeks of treatment in patients with active UC in a RDBPCT.^152^ Total bifidobacteria numbers on the mucosal surface of patients also increased, although it was not possible to determine if these were the administered bacteria or host bifidobacteria that benefited from the growth-promoting properties of the prebiotic. However, the authors concluded that a longer treatment period could lead to better clinical outcomes. Therefore, the same formulation was assessed in a RDBPCT where CD patients consumed the synbiotic for up to 6 months.^153^ Specifically, significant reductions in CD activity indices (*p* = 0.02) and histological scores (*p* = 0.018) were recorded for the synbiotic group. As for the previous study, mucosal-associated bifidobacteria increased in the test group. However, the authors noted that the synbiotic was most effective in patients with colon-related CD. The strain was later assessed alone for induction of remission in patients with active UC following 8 weeks of treatment in a multicentre RDBPCT.^154^ While 63% of patients in the test group showed remission, 52% in the placebo group also showed remission (*p* = 0.395) which is a very high percentage for a placebo group, as pointed out by the authors. This could be due to several trial design features, including ‘definition of remission’ but is most likely due to the standard medical treatments that all participants received. Despite this, a significant decrease was observed for UC disease activity index scores in the *B. longum* 536 group from baseline to week 8 (*p* < 0.01), but no significant decrease was observed in the placebo group (*p* = 0.88). Likewise, significant differences were observed in the 536 group from baseline to week 8 for the Rachmilewitz endoscopic index and the Mayo subscore but not in the placebo group. Mucosal healing rate was greater for the 536 group, but the difference between *B. longum* 536 and placebo groups was not significant.

The efficacy of VSL#3® for the treatment of UC has been investigated in several clinical trials. In combination with a low-dose prodrug of a conventional IBD treatment (balsalazide), VSL#3® proved significantly superior to conventional treatments alone (balsalazide or 5-aminosalicylic acid [5-ASA]) for obtaining remission in patients with active mild-to-moderate UC following 8 weeks of treatment.^155^ In patients with relapsing UC and receiving conventional 5-ASA treatment and/or immunosuppressants, VSL#3® treatment for 8 weeks was associated with significantly improved UC disease activity index scores compared with placebo (*p* = 0.01), improved rectal bleeding, and the formulation tended to induce remission in relapsing patients.^156^ As a sole treatment for mild-to-moderate active UC, VSL#3® resulted in significantly higher patient numbers with improved UC disease activity index scores following 6 weeks of administration compared with placebo (32.5 versus 10%, respectively, *p* = 0.001), and after 12 weeks of treatment, 42.9% of patients in receipt of VSL#3® achieved remission compared with 15.7% in the placebo group (*p* < 0.001).^157^ The formulation proved less effective for preventing CD recurrence, however, in patients following surgery in a multicentre RDBPCT following 90 d of treatment.^158^ However, the low rate of recurrence in the placebo group rendered this trial underpowered to observe statistical differences. In the second phase of the trial (an open-label study; days 91 to 365), patients receiving VSL#3® for the entire 365 d exhibited a lower rate of recurrence and lower levels of inflammatory cytokines though statistical significance was not observed.

#### Irritable bowel syndrome

Microbial formulations containing *B. longum* have also proven clinically effective for providing relief from IBS symptoms in adults (Table 2). Ki Cha et al.^159^ investigated the impact of a seven-strain species mix on diarrhea-dominant IBS that consisted of *B. longum, L. acidophilus, L. plantarum, L. rhamnosus, B. breve, B. lactis*, and *S. thermophilus* following daily treatment for 8 weeks. Throughout the study period, the proportion of participants in the intervention group reporting adequate relief from IBS symptoms was significantly higher than that in the placebo group (*p* < 0.05); however, the formulation failed to induce “superior effects” on individual symptoms including abdominal pain. The intervention significantly improved stool consistency and IBS quality-of-life improvement tended to be higher for the intervention group. Denaturing gradient gel electrophoresis profiles of the fecal microbiota revealed that the intervention was associated with stabilization of the intestinal microbiota. It should be noted that the follow-up period of 2 weeks is substantially less than the recommended follow-up for IBS trials of 6–12 months.

**Table 2.** An overview of clinical trials investigating the impact of *B. longum* on adults.

Condition/Disease/Biological Parameter	Participants; Age	Formulation	CFU; Dose; Duration	Clinical Effects and Biological Observations of Intervention Group Compared with Placebo Group	Reference; Trial ID
***Gastrointestinal Conditions***					
Ulcerative colitis	16; > 18 y	*B. longum* 536, Synergy 1 prebiotic (FOS & inulin)	2 x 10^11^, 6 g prebiotic; 2 daily doses; 4 weeks	Sigmoidal scores significantly reduced; β-defensins mRNA significantly reduced; TNF-α & IL1α significantly reduced; Reduced inflammation in biopsies; Regeneration of epithelial tissue	Furrie et al.^152^
Crohn’s Disease	35; > 18 y	*B. longum* 536, Synergy 1 prebiotic (FOS & inulin)	2 x 10^11^, 6 g prebiotic; 2 daily doses; 6 months	Significant reductions in CD activity indices & histological scores; TNF-α significantly reduced; Mucosal bifidobacteria increased	Steed et al.^153^
Ulcerative colitis	56; > 18 y	*B. longum* 536	2–3 x 10^11;^ 3 daily doses; 8 weeks	Significant decrease in UC disease activity index scores from baseline to week 8; Significant decrease in Rachmilewitz score from baseline to week 8; Significant decrease in Mayo subscore from baseline to week 8; Increased mucosal healing rate	Tamaki et al.^154^ (Multicentre)
Ulcerative colitis	90; > 18 y	VSL#3: *B. longum*, *B. breve*, *B. infantis*, *L. paracasei*, *L. plantarum*, *L. acidophilus*, *L. delbrueckii* subsp. *bulgaricus*, *S. salivarius* subsp. *thermophilus*, Balsalazide	9 x 10^11^, 2.25 g daily; 8 weeks	Significantly increased number of patients in remission; Achieved remission faster	Tursi et al.^155^
Ulcerative colitis	131; > 18 y	VSL#3: *B. longum*, *B. breve*, *B. infantis*, *L. paracasei*, *L. plantarum*, *L. acidophilus*, *L. delbrueckii* subsp. *bulgaricus*, *S. salivarius* subsp. *thermophilus*	3.6 x 10^14^, daily; 8 weeks	Significantly higher proportion of patients experienced improvement in UCDAI score of at least 50%; Achieved remission faster; Significantly improved rectal bleeding	Tursi et al.^156^ (Multicentre)
Ulcerative colitis	147; > 18 y	VSL#3: *B. longum*, *B. breve*, *B. infantis*, *L. paracasei*, *L. plantarum*, *L. acidophilus*, *L. delbrueckii* subsp. *bulgaricus*, *S. salivarius* subsp. *thermophilus*	3.6 x 10^12;^ 2 daily doses; 12 weeks	Significantly higher proportion of patients experienced improvement in UCDAI score of > 50%; Significantly increased number of patients in remission	Sood et al.^157^ (Multicentre)
Crohn’s Disease	119; > 16 y	VSL#3: *B. longum*, *B. breve*, *B. infantis*, *L. paracasei*, *L. plantarum*, *L. acidophilus*, *L. delbrueckii* subsp. *bulgaricus*, *S. salivarius* subsp. *thermophilus*	9 x 10^11;^ 2 daily doses; 365 d or beginning day 91 until day 365	Reduced mucosal inflammatory cytokine levels at days 90 & 365 for patients receiving probiotic for 365 d; Lower rate of recurrence among patients receiving probiotic for 365 d	Fedorak et al.^158^ NCT00175292 (Multicentre)
Irritable bowel syndrome	50; 18–65 y	*B. longum*, *L. acidophilus*, *L. plantarum*, *L. rhamnosus*, *B. breve*, *B. lactis*, *S. thermophilus*	1 x 10^10;^ daily; 8 weeks	Adequate relief significantly higher; Stool consistency significantly improved; IBS quality of life tended to be higher; Stabilisation of intestinal microbiota	Ki Cha et al.^159^
Irritable bowel syndrome	25; > 18 y	*B. longum* BB536, *L. rhamnosus* HN001, Vitamin B6	5 x 10^9^, 1.4 mg; daily; 1 month	Significantly improved abdominal pain, bloating & disease severity; Significantly improved colonic permeability; Increased lactic acid bacteria & bifidobacteria	Bonfrate et al.^160^ NCT03815617
Irritable bowel syndrome (diarrhea-dominant)	80; > 18 y	*B. longum* DSMZ 32,946, *B. bifidum* DSMZ 32,403, *B. lactis* DSMZ 32,269, *L. acidophilus* DSMZ 32,418, *L. rhamnosus* FloraActive™ 19070–2 FOS	5 x 10^9^, 947 mg; 2 daily doses; 8 weeks	Significant improvement in symptom scores	Skrzydlo-Radomańska et al.^161^ NCT04206410
Irritable bowel syndrome (IBS-C, IBS-D, IBS-M)	248; > 18 y	*B. longum* R0175	1 x 10^10;^ daily; 8 weeks	Improved quality of life in emotional wellbeing & social functioning; Increased energy levels that impacted willingness & ability to perform everyday tasks	Lewis et al.^162^ NCT02213172
Irritable bowel syndrome	362; > 18 y	*B. infantis* 35624	1 x 10^6^ or 1 x 10^8^ or 1 x 10^10;^ 4 weeks	1 x 10^8^ CFU significantly improved abdominal pain, bloating, bowel dysfunction, incomplete evacuation, straining, & flatulence	Whorwell et al.^163^
Irritable bowel syndrome	77; > 18 y	*B. infantis* 35624	1 x 10^10;^ 8 weeks	Significant reduction in symptom scores for abdominal pain/discomfort, bloating/distension, & bowel movement difficulty; Normalisation of abnormal IL-10/IL-12 ratio	O’ Mahony et al.^164^
Acute-radiation induced diarrhea	246; > 18 y	*B. longum* BB-536, *L. acidophilus* LAC-361	1.3 x 10^9^, 2 daily doses or 1 x 10^10^, 3 daily doses	No effect during radiation treatment in non-surgery patients but 1.3 x 10^9^ CFU reduced number of patients with moderate to severe diarrhea after radiation therapy; In surgery patients (before radiation therapy), 1.3 x 10^9^ CFU increased proportion of patients without very severe diarrhea during treatment.	Demers et al.^165^ NCT01839721
Constipation	94; > 18 y	*B. longum* UABI-14, *B. animalis* ssp. *lactis* UABIa-12, *B. bifidum* UABb-10, *L. acidophilus* DDS-1	1.5 x 10^10^, 1 daily dose	Faster normalisation of stool frequency & consistency after 1 week of treatment; Higher relative abundance of *Ruminococcaceae* & lower relative abundance of *Erysipelotrichaceae*	Martoni et al.^166^ NCT02418507
Lactose intolerance	23; > 18 y	*B. longum* BB536, *L. rhamnosus* HN001, Vitamin B6	5 x 10^9^, 1.4 mg; daily; 1 month	Significantly decreased bloating & improved constipation; Enriched genera involved in lactose digestion; Increased acetic acid, 2-methylpropanoic acid, nonenal, indolizine 3-methyl, decreased phenol	Vitellio et al.^167^ NCT03815617
Stress-induced gastrointestinal symptoms	75; > 18 y	*B. longum* Rosell-175, *L. acidophilus* Rosell-52	3 x 10^9;^ daily; 3 weeks	Significantly reduced abdominal pain & nausea/vomiting	Diop et al.^168^
***Immunity***					
Blood anti-oxidative activity in asymptomatic *H. pylori* colonised subjects	53; 20–60 y	*B. longum* 46, *L. paracasei* 8700:2, *L.* *fermentum* ME-3, FOS	3 x 10^9^, 6.6 g, 2 doses daily; 3 weeks	Significantly increased total antioxidative status; Significantly decreased ratio between oxidised & reduced glutathione	Hütt et al.^169^
Ulcerative colitis/Inflammatory biomarkers	22; 18–75 y	*B. infantis* 35624	1 x 10^10^, daily, 6 weeks	Significantly reduced C-reactive protein Numerically reduced IL-6	Groeger et al.^170^
Chronic fatigue syndrome/Inflammatory biomarkers	48; 18–65 y	*B. infantis* 35624	1 x 10^10^, daily, 8 weeks	Significantly reduced C-reactive protein & TNF-α Numerically reduced IL-6	Groeger et al.^170^
Psoriasis/Inflammatory biomarkers	26; 18–60 y	*B. infantis* 35624	1 x 10^10^, daily, 8 weeks	Significantly reduced C-reactive protein & TNF-α	Groeger et al.^170^
Healthy subjects	35; 18–65 y	*B. infantis* 35624	1 x 10^10^, daily, 8 weeks	No impact on pro-inflammatory markers	Groeger et al.^170^
Peritoneal dialysis patients/Inflammatory biomarkers & endotoxin	39; ≥ 18 y	*B. longum* A101, *B. bifidum* A218, *B. catenulatum* A302, *L. plantarum* A87	4 x 10^9^, daily; 6 months	Significantly reduced IL-6, TNF-α, IL-5, & endotoxin; Significantly increased IL-10 levels; Preserved residual renal function which significantly declined in the placebo group after 6 months.	Wang et al.^171^
Hemodialysis patients/Inflammatory biomarkers, endotoxin, & anti-heat shock protein 70 antibodies	75; 30–65 y	*B. longum* LAF-5, *B. lactis* BIA 6, *B. bifidum* BIA 6, *L. acidophilus* T16 FOS GOS Inulin	1.35 x 10^8^, 15 g, 4 times daily; 12 weeks	Significantly decreased pro-inflammatory markers (C-reactive protein & IL-6), endotoxin levels, & anti-heat shock protein 70 antibodies	Haghighat et al.^172^ IRCT2017041233393N1
***Cardiovascular Health***					
Normal or moderately elevated cholesterol	34; 18–65 y	*B. longum* BB536, *L. acidophilus* 145 in fermented milk drink	2.7x10^7^ −1x10^8^ (CFU/g) 1.4–2.1x10^8^ (CFU/g) 375 g daily; 4 weeks	Significantly decreased LDL-cholesterol in those with baseline level of total cholesterol > 190 mg/dl Significantly reduced HDL-cholesterol	Andrade and Borges,^173^
Type 2 diabetes	54; 35–70 y	*B. longum*, *L. acidophilus*, *L. casei*, *L. rhamnosus*, *L. bulgaricus*, *B. breve*, *S. thermophilus*	7 x 10^9^ 2 x 10^9^ 7 x 10^9^ 1.5 x 10^9^ 2 x 10^8^ 2 x 10^10^ 1.5 x 10^9^, daily; 8 weeks	Prevented increase in fasting plasma glucose, Significantly reduced serum hs-CRP, Increased plasma levels of glutathione	Asemi et al.^174^
***Depression, Anxiety, and Cognitive Functioning***					
Healthy/Psychological impact	55; 30–60 y	*B. longum* R0175, *L. helveticus* R0052	3 x 10^9^ daily; 30 d	Significantly alleviated psychological distress; Significantly reduced urinary cortisol	Messaoudi et al.^175^
Low mood for at least 2 y	79; ≥ 16 y	*B. longum* R0175, *L. helveticus* R0052	3 x 10^9^ daily; 8 weeks	No effect on low mood; No impact on inflammatory biomarkers	Romijn et al.^176^ ACTRN12613000438752
Major depressive disorder but on antidepressant drugs	81; 36.5 ± 8.03 y	*B. longum* R0175, *L. helveticus* R0052	≥10^10^ daily; 8 weeks	Significant decrease in the Beck Depression Inventory; Significant decrease in kynurenine/tryptophan ratio; Improved appetite	Kazemi et al.^177^ (IRCT2015092924271N1) Kazemi et al.^178^
Healthy/Cognition, Mood, Sleep quality	38; 18–35 y	*B. longum* BL04, *L. fermentum* LF16, *L. rhamnosus* LR06, *L. plantarum* LP01	4 x 10^9^ daily; 6 weeks	Significantly improved mood & sleep quality; Reduced depressive mood state, anger, & fatigue	Marotta et al.^179^ NCT03539263
IBS/Mild to moderate depression & anxiety	44; 26–58 y	*B. longum* NCC3001	1 x 10^10^ daily; 6 weeks	Significantly reduced depression scores; Significantly improved general physical health; Decreased brain responses to negative emotional stimuli based on fMRI analysis	Pinto-Sanchez et al.^180^ NCT01276626
Alzheimer’s disease/Cognitive functions & Metabolic status	79; 68–84.3 y	*B. longum*, *B. bifidum*, *L. acidophilus* & *selenium*	2 x 10^9^ 2 x 10^9^ 2 x 10^9^ 200 μg daily; 12 weeks	Significantly improved cognitive function; Significantly reduced hs-CRP, insulin levels, homeostasis model of assessment-insulin resistance, serum triglycerides; Significantly increased total antioxidant capacity, the quantitative insulin sensitivity check index; Significantly improved cholesterol profiles	Tamtaji et al.^181^ IRCT20170612034497N5
***Respiratory Health and Seasonal Allergies***					
Japanese cedar pollinosis during pollen season	44; 22–57 y	*B. longum* BB536	5 x 10^10;^ twice daily; 13 weeks	Significantly relieved symptoms; Significantly modulated Th2-skewed immune response; Suppressed *Bacteroides fragilis* levels in faecal microbiota	Xiao et al.^182^ Odamaki et al.^183^
Japanese cedar pollen exposure in environmental exposure unit	24; 25–56 y	*B. longum* BB536	5 x 10^10;^ twice daily; 4 weeks	Significantly reduced ocular symptom scores; Reduced disruption of normal activities following exposure; Significantly reduced total medications to alleviate symptoms	Xiao et al.^184^
Seasonal allergies during allergy season	173; 27 ± 1 year	*B. longum* MM2, *B. bifidum* G9–1, *L. gasseri* KS-13	3 x 10^9;^ daily; 8 weeks	Significantly improved rhinoconjunctivitis-specific quality of life	Dennis-Wall et al.^185^ NCT02349711
Perennial allergic rhinitis	95; 19–65 y	*B. longum* IM55, *L. plantarum* IM76	1 x 10^10;^ daily; 4 weeks	Significantly reduced total nasal symptom scores & rhinorrhea Numerically reduced sneezing & nasal congestion Significantly improved anti-allergic immunological profiles	Kang et al.^186^ KCT0003760
Common cold	454; adults	*B. longum* SP 07/3, *B. bifidum* MF 20/5, *L. gasseri* PA 16–8	5 x 10^7;^ daily; 3 months & 5.5 months (two different study groups)	Shortened common cold episodes by at least two 2 d & reduced severity of symptoms; After 14 d of supplementation, CD4+ T helper cells increased & cytotoxic plus T suppressor cells (CD8+) significantly increased	De Vrese et al.^187^
***Skin***					
Reactive skin	66; healthy females	*B. longum* lysate	10% lysate applied to skin twice daily; 2 months	Significantly decreased skin sensitivity; Reduced skin dryness	Guéniche et al.^188^

In the crossover RDBPCT, consumption of *B. longum* BB536 and *L. rhamnosus* HN001 with vitamin B6 for 1 month significantly improved IBS symptoms (abdominal pain and bloating) and disease severity compared with placebo (*p* < 0.0001).^160^ The formulation was also associated with improved colonic permeability as measured by sucralose recovery in urine – but had no impact on small intestinal permeability. Presumptive lactic acid bacteria and bifidobacteria increased during treatment, as did volatile organic compounds including butanoic, pentanoic, and propanoic acids, and hydrocarbons, while phenol decreased. However, the sample size of this crossover study was small at *n* = 25, and the washout period was 15 d, so a carryover effect of treatment cannot be ruled out.

A synbiotic preparation consisting of FOS and five bacterial strains including *B. longum* DSMZ 32,946, *B. bifidum* DSMZ 32,403, *B. lactis* DSMZ 32,269, *L. acidophilus* DSMZ 32,418, and *L. rhamnosus* FloraActive™ 19070–2 significantly improved diarrhea-associated IBS (IBS-D) symptoms in patients in an 8-week treatment period.^161^ Specifically, compared with the placebo, the synbiotic was associated with significant improvements on the IBS-Global Improvement Scale (−GIS) (*p* = 0.043) and the IBS-Symptom Severity Scale (−SSS) (*p* = 0.042) following 4 and 8 weeks of consumption. However, while 80 patients were enrolled in the study, only 68 completed it, thus the sample size was relatively small such that the statistical power necessary to determine statistically significant differences between treatments could be limited. Also, the authors cannot rule out if the maltodextrin administered to the placebo group (but not the synbiotic group) had an impact given that IBS severity significantly decreased from baseline in the placebo group after weeks 4 and 8 of the trial. Thus, further trials are warranted with larger sample sizes and a better placebo treatment to enable a more accurate comparison of placebo and intervention.

The impact of *B. longum* R0175 alone on the gastrointestinal symptoms and psychiatric comorbidities of IBS was investigated by Lewis et al.^162^ in a 3-arm RDBPCT involving 251 adults with either constipation-associated IBS (IBS-C), IBS-D, or mixed pattern IBS (IBS-M). The other strain that was assessed was *L. paracasei* HA-196, and the treatment period lasted for 8 weeks. Questionnaires were used to assess IBS symptoms, stool consistency, frequency, and quality of life. *L. paracasei* proved to be the most effective strain for improving IBS symptoms, particularly for IBS-C and IBS-D patients. IBS patients are also at increased risk of depression, anxiety, bipolar, and sleep disorders.^189^ Both strains improved emotional well-being and social functioning, but participants consuming *B. longum* R0175 also reported increased energy levels that positively impacted their willingness and ability to perform everyday tasks. A placebo effect of 33% was recorded in this study. A meta-analysis conducted by Patel et al.^190^ reported that the placebo response in participants with IBS can range from 16% to 71% for pharmaceutical interventions or natural health products. Lewis et al.^162^ suggested that prior to the intervention, a longer period examining bowel habits of participants may help to mitigate the placebo effect.

Whorwell et al.^163^ reported that administered bacterial dose can significantly impact efficacy. In a study involving 362 primary care IBS patients, *B. infantis* 35624 failed to provide relief from symptoms at doses of 10^6^ and 10^10^ colony forming units (CFU)/ml for 4 weeks but at 10^8^ CFU/ml the strain significantly relieved many IBS symptoms compared with placebo (and other doses) including abdominal pain, as well as bloating, bowel dysfunction, flatulence, straining, and incomplete evacuation. In this case, the strain was prepared as a freeze-dried powder and packed into capsules with an excipient. A previous study had demonstrated the efficacy of 10^10^ CFU/ml of *B. infantis* 35624 for relieving IBS symptoms in patients when administered in a milk drink – a response that was associated with a normalization of the anti-inflammatory to pro-inflammatory cytokine ratio.^164^ In capsule form, the 10^10^ CFU/ml of *B. infantis* 35624 ‘coagulated’ into a ‘glue-like mass’ which the authors state is due to the intense hygroscopic nature of the strain that presumably impacted its growth characteristics in the gut; but with time the higher formulation should lead to noticeable benefits as the bacterium replicates to the concentration required to achieve efficacy.

#### Diarrhea

Diarrhea is a common side effect of pelvic radiation therapy with up to 80% of patients reported suffering from acute radiation-induced diarrhea.^191^ *B. longum* has proven effective in the treatment of diarrhea when used in combination with other strains. FloraActive™ is a formulation containing *B. longum* BB-536 and *L. acidophilus* LAC-361. In a RDBPCT, 246 pelvic radiation patients were randomized to receive a placebo or one of the two doses of FloraActive™; a standard dose twice daily (1.3 × 10^9^ CFU) or a high dose, thrice daily (1 × 10^10^ CFU).^165^ Patients began taking the formulation in capsule form on the first day of therapy and continued until treatment ended with time to first appearance of grade≥2-3-4 diarrhea using Kaplan–Meier curves. The formulation did not prevent moderate-to-severe diarrhea during treatment. However, at the end of treatment or during the 2 weeks after treatment, 35% of the standard dose group experienced less of the moderate-to-severe diarrhea compared with only 17% in the placebo group (*p* = 0.04). In patients who had surgery before radiation commenced, the standard dose group had a higher proportion of patients without very severe (grade 4) diarrhea (97%) versus placebo (74%) (*p* = 0.03) during treatment. The higher dose formulation proved less effective than the standard dose, highlighting again the importance of clinical data at different dosage levels. It should be noted that a dietary intervention was included in this study for all participants based on individualized recommendations by a dietitian that generally involved reducing total intake of lipids and advice on intake of dietary fibers and simple or complex carbohydrates; this may have reduced digestive symptomatology in all participants.

#### Constipation

The prevalence of functional constipation amongst adults in the community has been estimated to be 14%.^192^ In a RDBPCT, a formulation with *B. longum* and three other strains (*B. animalis* ssp. *lactis*, *B. bifidum*, *L. acidophilus*) failed to achieve improvements in symptomology (based on patient assessment of constipation – symptom [PAC-SYM] and patient assessment of constipation – quality of life [PAC-QoL]) compared with the placebo group.^166^ The authors state that this was possibly due to a high placebo response, which can be the case for participants with bowel disorders but may be controlled with a placebo run-in period that would enable the exclusion of high responders. Also, the PAC-SYM score was not included in the initial inclusion criteria, which again may have impacted the ability to differentiate between groups at the end of the trial. Despite this, the formulation was associated with faster normalization of stool frequency and consistency following 1 week of treatment in the 4-week intervention period. The study assessed fecal microbiota at baseline and endpoint of the study and reported a significantly higher relative abundance of *Ruminococcaceae* and lower relative abundance of *Erysipelotrichaceae* in the formulation group. Interestingly, the abundance of the *Ruminococcaceae* family has been shown to positively correlate with faster intestinal transit and improved Bristol Stool Scale scores.^193^

#### Lactose intolerance

It has already been demonstrated that the formulation *B. longum* BB536 and *L. rhamnosus* HN001 with vitamin B6 proved effective for the treatment of IBS.^160^ The same formulation also alleviated the symptoms of lactose intolerance in lactose-intolerant subjects following 30 d of treatment in a crossover RDBPCT.^167^ The formulation was associated with increased fecal abundance of lactose-digesting genera including *Bifidobacterium* that positively correlated with increased acetic acid and 2-methylpropanoic acid, while decreased phenol positively correlated with relative amounts of genera from Proteobacteria. A previous study found that growing *B. longum* B6 in lactose prior to treatment increased its ability to improve lactose digestion in sufferers as it induces higher β-galactosidase activity in the strain.^194^

#### Stress-induced gastrointestinal symptoms

Psychological stress is known to contribute to several gastrointestinal dysfunctions through the brain-gut axis.^195^ The formulation Probio-Stick which contains *B. longum* Rosell-175 and *L. acidophilus* Rosell-52 proved effective in relieving stress-induced gastrointestinal symptoms in volunteers in a RDBPCT following 3 weeks of intervention.^168^ Specifically, the formulation significantly reduced abdominal pain (*p* = 0.004) and nausea/vomiting (*p* = 0.009) in the volunteers who had been selected for the trial based on suffering from daily stress with at least two stress-induced symptoms in the previous month. However, the sample size was small at *n* = 23 suggesting that further trials are warranted with greater participant numbers to confirm these results. For further details of these trials, see Table 2.

### Immunity

*Helicobacter pylori* infection of the stomach causes persistent oxidative stress in the stomach and induces chronic inflammation that can lead to peptic ulcers, gastritis, and gastric cancer.^196,197^ Hütt et al.^169^ investigated the impact of a synbiotic product on antioxidative activity in asymptomatic subjects, 53% of which were colonized with *H. pylori* in a cross-over RDBPCT. Blood sera samples were analyzed for total antioxidative status (TAS) and de-proteinated whole blood, plasma, and erythrocyte lysate were analyzed for oxidized glutathione (GSSG) and reduced glutathione (GSH). The synbiotic consisted of Raftilose P95® (oligofructose) and three bacterial strains, namely *B. longum* 46, *L. fermentum* (now *Limosilactobacillus fermentum*) ME-3, and *L. paracasei* 8700:2. The *L. fermentum* strain had previously demonstrated high TAS while all three strains exhibited moderate antagonistic activity against *H. pylori*.^198^ The *H. pylori*-colonized subjects had significantly reduced sera levels of TAS compared with *H. pylori*-negative subjects. Following 3 weeks of synbiotic administration, TAS values in *H. pylori*-colonized subjects significantly increased compared with baseline (*p* = 0.004) and the ratio between oxidized and reduced glutathione decreased (*p* = 0.016). There was no impact on *H. pylori* colonization, but the authors suggest that the enterocoated synbiotic capsules were only soluble in the small intestine and thus the bacterial strains did not encounter *H. pylori* in the stomach.^169^

In three separate RDBPCTs, the strain *B. infantis* 35624 was evaluated for its impact on inflammatory biomarkers in patients with UC, chronic fatigue syndrome, and psoriasis, the latter two being examples of extra-intestinal inflammatory diseases.^170^ The impact of bacterial strain on inflammatory biomarkers of healthy subjects was also assessed. Inflammatory biomarkers included C-reactive protein (CRP), and the pro-inflammatory cytokines IL-6 and TNF-α. The serum or plasma level of the acute phase protein, CRP, is a useful indicator of systemic pro-inflammatory activity in several inflammatory conditions.^199^ Compared with healthy volunteers, all patients exhibited significantly increased levels of CRP (*p* < 0.001), IL-6 (*p* < 0.05), and TNF-α (*p* < 0.001) at baseline. In UC patients, consumption of the strain for 6 weeks was associated with significantly reduced CRP levels compared with the placebo group (*p* = 0.0327), while the reduction in IL-6 levels approached significance (*p* = 0.057). The strain had no impact on TNF-α levels in UC patients. For those suffering from chronic fatigue syndrome, 8 weeks of oral administration of the strain significantly reduced CRP (*p* = 0.0285) and TNF-α (*p* = 0.0214) levels compared with the placebo group, while the reduction in IL-6 levels approached significance (*p* = 0.054). Psoriasis patients also consumed the strain for 8 weeks where it was associated with significant reductions in CRP (*p* = 0.0425) and TNF-α (*p* = 0.0405) levels but had no impact on IL-6. The strain had no impact on the pro-inflammatory biomarkers in healthy individuals following 8 weeks of administration compared with placebo. However, lipopolysaccharide-stimulated peripheral blood mononuclear cells from *B. infantis* 35624-treated healthy subjects demonstrated significantly reduced *in vitro* secretion of TNF-α and IL-6. The authors state that the reductions in inflammatory markers observed in this trial would be indicative of clinical remission and a lower risk of relapse but larger patient numbers would be necessary to demonstrate clinical efficacy.

Chronic kidney disease is associated with chronic inflammation and thus elevated levels of IL-6 and TNF-α.^171^ Endotoxin, from the outer membrane of gram negative bacteria, is both a source and indicator of inflammation in this disease.^200,201^ Furthermore, the gut microbiota of these patients is disrupted by dramatic reductions in commensal species, such as bifidobacteria and lactobacilli.^202^ In a RDBPCT, Wang et al.^171^ investigated the impact of a formulation on cytokine and endotoxin levels in peritoneal dialysis patients. The formulation contained *B. longum* A101, *B. bifidum* A218, *B. catenulatum* A302, and *L. plantarum* A87 and was administered to patients for 6 months. Patients in the intervention group displayed significantly reduced IL-6 (*p* < 0.001), TNF-α (*p* = 0.019), IL-5 (*p* = 0.002), and endotoxin (*p* = 0.007) by the end of treatment and significantly increased IL-10 levels (*p* = 0.027) compared with placebo. While the residual renal function significantly declined in the placebo group (*p* = 0.008) after 6 months, residual renal function was preserved in the intervention group after the same time period (*p* = 0.176). However, there were no significant differences between groups for clinical outcomes, such as cardiovascular events and peritonitis. This study was conducted on a small patient number (*n* = 39). Also, the lack of long-term follow-up is a limitation of the study given the chronic nature of the disease. Therefore, trials with larger sample sizes and longer-term follow-ups that focus on other parameters, such as cardiovascular events – given that CVDs are responsible for approximately 50% of all deaths at end-stage renal disease – are warranted to confirm the clinical usefulness of this formulation for chronic kidney disease patients.

In hemodialysis patients, a synbiotic formulation proved even more effective than the bacterial formulation alone for significantly decreasing pro-inflammatory markers (C-reactive protein and IL-6), endotoxin levels, and anti-heat shock protein 70 antibodies compared with the placebo following 12 weeks of intervention in a RDBPCT.^172^ Heat shock protein 70 protects cells from urea-induced damage.^203^ The synbiotics consisted of FOS, galactooligosaccharides (GOS), and inulin with *B. longum* LAF-5, *B. lactis* BIA 6, *B. bifidum* BIA 6, and *L. acidophilus* T16, while the bacterial formulation alone consisted of the same four strains. The authors state that the short timeline of the study (12 weeks) did limit the statistical power for detection of parameter changes in the two groups.

For further details of these trials, see Table 2.

### Cardiovascular health

#### Blood lipid profiles

In a crossover RDBPCT, daily consumption of a fermented milk drink containing *B. longum* BB536 (known to have high bile salt hydrolase activity^204^) and *L. acidophilus* 145 by women with normal or moderately elevated cholesterol significantly decreased LDL-cholesterol (*p* = 0.014) in those with a baseline level of total cholesterol above 190 mg/dl.^173^ The authors suggest that this result may be due to the genotype of apolipoprotein E of participants, which is known to impact the response to dietary lipids. High-density lipoprotein (HDL) cholesterol was also significantly reduced (*p* < 0.01) by the BB536-containing fermented milk, which may increase the risk for CVDs, thus this formulation requires further testing and specific mechanisms of action need to be unraveled.

#### Type 2 diabetes

Type 2 diabetes is a known risk factor for CVD with patients presenting with dyslipidaemia along with insulin resistance. Increased levels of inflammation and oxidative stress have also been reported in type 2 diabetic patients.^205^ Asemi et al.^174^ presented the first trial investigating the impact of a bacterial formulation on metabolic profiles, high-sensitivity- (hs-) CRP, and oxidative stress in type 2 diabetic patients. The inclusion criteria included fasting plasma glucose ≥126 mg/dl, blood sugar (2 h postprandial) ≥200 mg/dl, and hemoglobin A1 C (HbA1 C) ≥6.5%. The multispecies mix consisted of seven different strains including *B. longum*, *L. acidophilus*, *L. casei* (now *Lacticaseibacillus casei*), *L. rhamnosus*, *L. bulgaricus*, *B. breve*, and *S. thermophilus*. After 8 weeks of supplementation, the formulation prevented an increase in fasting plasma glucose compared with the placebo (*p* = 0.01). It was also associated with significantly reduced serum hs-CRP (*p* = 0.02) and increased plasma levels of the antioxidant glutathione (*p* = 0.03). However, it had no impact on lipid profiles, serum insulin, uric acid, and total antioxidant capacity. It would be interesting to determine if higher doses of the formulation could impact these parameters.

For further details of these trials, see Table 2.

### Depression, anxiety, and cognitive functioning

#### Anxiety and depression

According to the WHO, over 300 million people globally suffer from depression and an estimated 264 million people suffer from anxiety disorders.^206^ Existing pharmacological treatments tend to vary in terms of efficacy with many exerting only modest effects.^207^ An increasing number of studies are investigating the efficacy of beneficial bacteria as treatments for anxiety and depressive disorders.

The formulation *B. longum* R0175 and *L. helveticus* R0052 was shown to significantly reduce anxiety-like behavior in rats (*p* < 0.05) following 2 weeks of treatment based on a screening model for anti-anxiety agents.^175^ In the same study, the formulation was assessed for its psychological impact on normal volunteers following 30 d of administration in a RDBPCT. In humans, the bacterial formulation alleviated psychological distress with volunteers showing lower scores for somatization (*p* < 0.05), depression (*p* < 0.05), and anger-hostility as evaluated via the Hopkins Symptom Checklist (HSCL-90), and lower scores for the Hospital Anxiety and Depression Scale (HADS global score, *p* < 0.05; HADS anxiety score, *p* < 0.06). Test subjects also performed better at problem-solving as measured via the Coping Checklist CCL) (*p* < 0.05). While urinary cortisol levels remained stable in the placebo group, levels in the intervention group decreased over time (*p* < 0.05). However, the same formulation had no impact on participants with low mood in a RDBPCT following 8 weeks of treatment when used as a primary treatment.^176^ The formulation also had no impact on inflammatory biomarkers. The participants in this trial reported continuous low mood for at least 2 y, thus the authors suggest that the bacterial strains may be more effective for treating patients reporting shorter terms of low mood or less severe low mood, or that its use as a primary treatment may take longer than 8 weeks to exert its effects. In line with this, a more recent trial investigating the same strains in patients with major depressive disorder in receipt of antidepressant drugs for at least 3 months before the trial reported a significant decrease in the Beck Depression Inventory (BDI) score following 8 weeks of treatment compared with placebo (*p* = 0.042).^177^ The formulation was also associated with a significant decrease in the kynurenine/tryptophan ratio (*p* = 0.048) thus impacting serotonin levels by driving tryptophan along the serotonin pathway as opposed to its conversion to kynurenine.^208^ In a post hoc analysis of the same clinical trial, Kazemi et al.^178^ reported that the bacterial strains were associated with improved appetite in major depressive disorder patients, an important finding given that poor appetite and weight loss can be features of depression. However, the study has limitations including the fact that due to the lengthy recruitment phase, the trial was conducted at different times/seasons of the year, which could have an impact on depression.^209^ The patients were not all taking the same antidepressant drug. Other differences between groups, including lifestyle, diet, and vitamin D status, may have diluted the effect of the formulation. Thus, confirmatory trials without these limitations are needed. It would also be of interest to determine if the bacterial strains in the formulation exert pharmacological effects on the antidepressant drugs.

The bacterial strains *B. longum* BL04, *L. fermentum* LF16, *L. rhamnosus* LR06, and *L. plantarum* LP01 were reported to significantly improve mood and sleep quality, and reduce depressive mood state, anger, and fatigue in healthy volunteers in a RDBPCT.^179^ However, the trial consisted of 38 participants, thus larger scale trials are warranted to confirm these results. Furthermore, the results of the study are based on a self-reporting questionnaire, thus, as the authors suggest, future confirmatory trials would benefit from “categorical diagnostic tools” and clinical assessments of participants.

Patients suffering from IBS often report comorbidities such as depression, anxiety, and somatization, amongst others.^210^ In this respect, *B. longum* NCC3001 was assessed for its efficacy to treat depression and anxiety in IBS patients following 6 weeks of administration in a RDBPCT.^180^ The strain was previously shown to normalize anxiety-like behavior in a mouse model of chemical colitis via the vagal pathway and it reduced excitability of enteric neurons.^211^ It was also shown to normalize the Brain-Derived Neurotrophic Factor (BDNF) in mice with mild-to-moderate colonic inflammation.^212^ BDNF has been identified as a link in the gut-brain axis,^213^ and reduced expression has been associated with gut dysbiosis and the onset of anxiety-like behavior in germ-free animals.^214^ By week 6, 14/22 patients in the NCC3001 group exhibited reduced depression scores of at least 2 points on the HAD scale, compared with 7/22 patients in the placebo group (*p* = 0.04).^180^ At week 10, depression scores were still reduced in the intervention group. Quality of Life in the physical subdomain also significantly increased for the intervention group (*p* = 0.03) with patients reporting improvements in general physical health. Functional magnetic resonance imaging (fMRI) analysis revealed that the bacterial strain was associated with decreased responses to negative emotional stimuli in various brain areas including the amygdala and fronto-limbic regions compared with placebo. Despite the promising results, the authors suggest that clinician-administered rating scales may be superior to the HAD scale used in this study. Furthermore, the placebo group showed lower baseline depression scores than the test group although the authors confirmed that after adjusting for baseline differences, the statistically significant result in favor of NCC3001 remained, as it did when only a subset of patients with baseline scores indicating depression (HAD-D ≥ 8) was analyzed. Thus, strategically designed confirmatory trials without these limitations are warranted to verify the data.

#### Alzheimer’s disease

Alzheimer’s disease is a progressive disease of the brain and is believed to contribute to 60–70% of dementia cases worldwide.^215^ Disease pathology is associated with the presence of β-amyloid plaques in the brain.^216^ However, several other factors have also been linked to disease pathogenesis, including the gut microbiota.^217,218^ Physiological features identified in the blood of Alzheimer’s disease patients include reduced antioxidant capacity and increased levels of reactive oxygen species (ROS), as well as increased markers of inflammation.^219^ Based on these observations, Tamtaji et al.^181^ performed a RDBPCT investigating the impact of three bacterial strains and the trace element selenium on cognitive function and metabolic status of patients with Alzheimer’s disease. Selenium has been shown to attenuate the pathology of Alzheimer’s disease and protect against cognitive decline.^220^ The bacterial strains included *B. longum*, *B. bifidum*, and *L. acidophilus* and patients were administered selenium alone or selenium co-supplemented with the bacteria, or placebo for 12 weeks.^181^ The combination of strains and selenium proved most effective as it was associated with improved cognitive function with a significant increase in the mini-mental state examination score in patients receiving co-supplementation compared with those on selenium alone or placebo (*p* < 0.001). Co-supplementation was also associated with an improvement in some metabolic profiles including significantly reduced hs-CRP (*p* < 0.001) and significantly increased total antioxidant capacity (*p* < 0.001), significantly lower insulin levels (*p* < 0.001), significantly lower homeostasis model of assessment-insulin resistance (HOMA-IR) (*p* < 0.001) and increased quantitative insulin sensitivity check index (QUICKI) (*p* < 0.006); it was associated with significantly decreased serum triglycerides (*p* = 0.02) and significantly improved (*p* < 0.05) cholesterol profiles. These findings are particularly relevant given the link between insulin resistance and Alzheimer’s disease,^221^ and the potential role of high triglyceride levels in cognitive impairment.^222^ It would be interesting to determine if the bacterial formulation alone is capable of exerting similar effects to the co-supplemented product, particularly since selenium supplementation has recently been touted as a “good alternative” to relieve some symptoms of mild cognitive impairment and Alzheimer’s disease.^223^ For further details of these trials, see Table 2.

### Respiratory illness

#### Seasonal allergy

In adults, *B. longum* BB536 has proven to be effective in the treatment of Japanese cedar pollinosis, an IgE-mediated type 1 allergy,^182,184^ which has been described as one of the most common allergic diseases in Japan.^224^ During 13 weeks of treatment with *B. longum* BB536 in subjects with a clinical history of Japanese cedar pollinosis (and during high pollen season), nine subjects out of 22 in the placebo group had to leave the trial prematurely to take prescribed medication as opposed to only 2 subjects out of 22 in the intervention group.^182^ Significant decreases in symptom scores for rhinorrhea (*p* = 0.0167) and nasal blockage (*p* = 0.0118) were recorded for the BB536 group compared with placebo and composite scores of the weekly scores for sneezing, rhinorrhea, nasal blockage, nasal itching, eye symptoms, and throat symptoms were significantly lower in the BB536 group (*p* = 0.0339). Pollen dispersion was associated with a Th2-skewed immune response. However, BB536 tended to suppress Japanese cedar pollinosis-specific IgE, and significantly suppressed elevations in plasma thymus- and activation-regulated chemokine (TARC) at weeks 4 (*p* = 0.038) and 8 (*p* = 0.031) compared with placebo. TARC has been used in several studies to measure disease severity in atopic dermatitis.^225^ In a follow-on study, fecal samples of the trial participants were investigated for changes to the gut microbiota.^183^ Subjects suffering from Japanese cedar pollinosis had increased levels of *Bacteroides fragilis* compared with healthy subjects, but this increase was suppressed by *B. longum* BB536 treatment. The same research group examined the impact of 4 weeks of BB536 intake on Japanese cedar pollen exposure in an environmental exposure unit (EEU) in a crossover RDBPCT.^184^ Ocular symptom scores were significantly reduced for the BB536 group compared with placebo during the 4 h of pollen exposure (*p* < 0.1), and scores for disruption of normal activities following exposure were also lower in the BB536 group. Furthermore, the BB536 group used significantly fewer total medications to alleviate symptoms compared with the placebo group (*p* = 0.041). However, in both trials, sample sizes were low with only 24 participants in the latter trial. Furthermore, in the latter trial, BB536 failed to impact other symptoms including nasal and throat symptoms. The authors suggest that differences in exposure patterns between the pollen season and the EEU could account for this where the latter involved a heavy and concentrated exposure for 4 h which may have been too aggressive for BB536 to alleviate symptoms. Thus, the EEU may not be a suitable model to verify treatment efficacy for seasonal allergies.

In adult participants who self-identified as having seasonal allergies, the bacterial formulation *B. longum* MM2, *B. bifidum* G9–1, and *L. gasseri* KS-13 was associated with a significant improvement in rhinoconjunctivitis-specific quality of life during allergy season when compared with the placebo group (*p* = 0.0092).^185^ Changes in the immune parameters tested (serum total IgE and Treg percentage), from baseline to week 6 were similar for both the intervention and placebo groups such that the mechanism of action remains unclear. The authors suggest that administration of the bacterial formulation before commencement of allergy season may have generated different results by allowing the gut microbiota and immune system time to respond to the intervention before allergen exposure. Further trials are warranted to determine if prophylactic administration of the formulation could improve its efficacy.

For sufferers of perennial allergic rhinitis, 4 weeks of consuming the formulation NVP-1703 composed of *B. longum* IM55 and *L. plantarum* IM76 significantly reduced total nasal symptom scores at weeks 1, 3, and 4.^186^ It also significantly reduced rhinorrhea at weeks 1, 3, and 4, and numerically, but not statistically, reduced sneezing and nasal congestion over the 4 weeks. In terms of immunity, NVP-1703 was associated with improved anti-allergic immunological profiles of participants by significantly increasing IL-10 levels (*p* = 0.047), and significantly increasing the ratios of IL-10/IL-14 (*p* = 0.046) and IL-10/IL-13 (*p* = 0.018). NVP-1703 was also associated with significantly reduced allergy-specific IgE levels (*p* = 0.033). The study also reported that NVP-1703 reduced urinary levels of prostaglandin F_2α_ and leukotriene E_4_, though not significantly. These molecules are useful biomarkers of eosinophil and mast cell activation – involved in allergic inflammation. The participants in this study suffered mild-to-moderate persistent rhinitis, capable of enduring symptoms without medication. Thus, it would be worthwhile investigating the efficacy of NVP-1703 in patients with more severe allergy and determine if the formulation is as effective as or better than conventional treatments.

#### Common cold

In terms of the common cold, consumption of the strains *B. longum* SP 07/3, *B. bifidum* MF 20/5, and *L. gasseri* PA 16–8 by two different cohorts of people in a RDBPCT during two winter/spring periods for at least 3 months resulted in a shortening of common cold episodes by at least 2 days and reduced severity of symptoms compared with the placebo group (*p* = 0.045).^187^ Fourteen days after supplementation, the strains were associated with increased CD4+ T-helper cells and significantly increased cytotoxic plus T-suppressor cells (CD8+) compared with placebo (*p* = 0.035) in a randomly tested subset of participants. Both CD4+ and CD8+ are involved in cell-mediated immunity, thus, they destroy infected cells.

For further details of these trials, see Table 2.

### Skin health

#### Reactive skin

A *B. longum* lysate (an ultrasound-inactivated suspension) applied to face, arm, and leg skin as a topical cream by healthy female volunteers with reactive skin for a 2-month intervention period was associated with a significant decrease in skin sensitivity by day 57 (*p* = 0.0024), whereby the skin exhibited increased resistance to physical and chemical aggression.^188^ Volunteers in the test group also reported a reduction in skin dryness after 29 d of applying the cream (*p* = 0.03). However, the lysate failed to improve skin barrier recovery rate. Based on an *ex vivo* human skin model, the lysate was found to decrease vasodilation, edema, mast cell degranulation, and TNF-α release, and in nerve cells, it reduced neuron reactivity and accessibility.

For further details of this trial, see Table 2.

## *B. longum* effects in elderly

### Gastrointestinal health and disease

Aging significantly impacts the composition and functionality of the gut microbiome,^226^ which has been attributed to several factors including lifestyle changes, ill health, and medication. A reduction in SCFA producers, including bifidobacteria, increased abundance of pathogens and facultative anaerobes, and a reduction in microbial diversity are recognized features of the elderly microbiome.^227–229^

### Constipation

Constipation can be a common issue for elderly people living in nursing homes. To investigate the efficacy of *Bifidobacterium* to improve bowel movement regularity in nursing home residents, Pitkala et al.^230^ enrolled 209 subjects in a RDBPCT where residents were randomized to receive either a fermented oat drink with 1 × 10^9^ CFU of two *B. longum* strains (46 and 2c) or the oat drink with *B. lactis* BB12 (1 × 10^9^ CFU) or the placebo oat drink without viable bacteria, daily for 7 months. Residents receiving bacterial strains had significantly more frequent bowel movements (without causing diarrhea) than those in the placebo group. The administration of these strains on elderly nursing home residents suffering from constipation should help improve quality of life. However, there were several limitations within this study due to the challenging nature of the study population. Indeed, a number of participants refused to cooperate during study follow-up, and a number of participants died during the study. Of the initial 209 participants, 179 completed the study. Indeed, the authors state that randomized intervention studies on frail nursing home residents are rare as a consequence of such challenges.

The administration of *B. longum* BB536 to elderly patients receiving enteral feeding for 16 weeks significantly normalized defecation frequency in subjects with low and high-frequency defecation.^231^ However, the authors state that the effects were mild when compared with other therapies, such as laxatives and prokinetics. Also, the authors caution against extrapolating the outcome to individuals with severe and/or chronic gastrointestinal issues.

### Immunity

As we age, organ function changes, a process referred to as senescence.^239^ Immunosenescence describes the immune dysfunction that occurs with aging^240^ and is associated with many factors, one of which is chronic inflammation.^239^ Indeed, pro-inflammatory cytokines have been shown to increase with aging.^241,242^ Given that the thymus degenerates with age and T cell production decreases, strategies that restore T cell production have been proposed as effective interventions for immunosenescence.^239^ The immune-modulating capabilities of bacteria, such as *B. longum* potentially render them ideal candidates to stimulate the immune system of older people. Thus, a number of RDBPCTs have investigated various commensal bacteria for their ability to beneficially modulate the immune response.

In the previous section on *Constipation*, the RDBPCT conducted by Pitkala et al.^230^ revealed that consumption of a fermented oat drink with *B. longum* strains 46 and 2c or *B. lactis* BB12 by elderly institutionalized residents significantly improved the frequency of bowel movements. A follow-on study investigated fecal bifidobacteria and two anti-inflammatory and one pro-inflammatory cytokine (TGF-β1, IL-10, TNF-α, respectively) in 55 of these subjects.^232^ While the study subjects were found to have relatively high levels of *Bifidobacterium* (10^10^ cells/g feces), consumption of the *B. longum* strains resulted in increases in *B. adolescentis* and *B. catenulatum* along with a modest increase in *B. longum*. The intervention did not significantly impact the serum cytokine levels, but significant associations were found between cytokine levels and the presence of specific *Bifidobacterium* species, which was observed for the two intervention groups (two *B. longum* strains 46 and 2c, or *B. lactis* BB12) and the placebo. Interestingly, lower levels of IL-10 were observed in the presence of members of the *B. longum* group, while the presence of *B. breve* correlated with higher levels of TGF-β1, although the changes were small, and the biological relevance of the results is uncertain.

Twelve weeks of supplementation with *B. longum* BB536 in hospitalized elderly patients receiving enteral tube feeding was associated with significantly increased fecal bifidobacteria.^233^ At weeks 4 and 16, serum IgA was increased in the intervention group compared with the placebo, though this was not significant. Interestingly, the significant decline in NK cell activity observed in the placebo group throughout the study was not observed in the BB536 group where NK cell activities were maintained at a stable level. Furthermore, the study found that a subgroup of participants with low initial NK cell activity particularly benefited from the bacterial strains, which increased NK cell activity from baseline throughout the study and was significantly different from the placebo at weeks 8 and 12. Other studies have reported that probiotics can improve innate immunity in participants with low NK cell activities.^243–245^ Furthermore, the BB536 group showed a tendency for increased number of bowel movements and lower body temperature.

In a RDBPC-crossover study involving healthy elderly subjects mainly recruited from the community, consumption of a synbiotic consisting of *B. longum* and the prebiotic Synergy 1 (FOS, inulin) for 4 weeks was associated with significantly increased fecal bifidobacteria counts (*p* < 0.0001), and Actinobacteria members (*Atopobium* group as well as bifidobacteria) (*p* = 0.0004) and Firmicutes (*p* < 0.0001) and significantly reduced Proteobacteria members (*p* < 0.0001).^234^ Butyrate production was also increased in the synbiotic group (*p* < 0.04). In terms of immunity, a number of cytokines were assessed, but of them all, the synbiotic only significantly impacted the levels of the pro-inflammatory cytokine TNF-α, which were reduced at weeks 2 (*p* = 0.02) and 4 (*p* = 0.0406). The synbiotic did not impact any of the measured clinical parameters including C-reactive protein, full blood counts, blood lipids, and levels of glucose, insulin, and immunoglobulins.

Three weeks of consuming a formulation consisting of *B. longum* MM2, *B. bifidum* G9–1, and *L. gasseri* KS-13 maintained CD4+ lymphocytes in elderly subjects and was associated with significantly increased IL-10 levels (*p* < 0.0001) compared with placebo, resulting in a less inflammatory cytokine profile in a crossover RDPCT.^235^ However, the crossover nature of the study was associated with limitations. First, during the second stage of the study, the time of year (start of cold and flu season) impacted the cytokine analysis as concentrations were highest at the final time point independent of the intervention or placebo. The washout period of 5 weeks was deemed too short since a carryover effect was observed for the cytokines in participants who had initially received the formulation but were then in the placebo group in period 2.   The intervention was found to modify the fecal microbiota with test subjects having significantly increasing bifidobacteria (*p* < 0.05), lactic acid bacteria (*p* < 0.05), and decreasing *E. coli* levels (*p* < 0.05). In a more recent RBDPCT, consumption of the formulation *B. longum* Bar33 and *L. helveticus* Bar13 by elderly subjects for 30 d was associated with significantly improved immune function via an increase in naïve, activated memory, regulatory T cells, B cells, and NK cells and a decrease in memory T cells (*p* < 0.05) compared with the placebo.^236^ In the same study, the authors studied the two strains in mice and revealed that they significantly increased regulatory T cells while decreasing γδ T cells, and increased B cells compared with control mice. For further details of these trials, see Table 3.

**Table 3.** An overview of clinical trials investigating the impact of *B. longum* on elderly.

Condition/Disease/Biological Parameter	Participants; Age	Formulation	CFU; Dose; Duration	Clinical Effects and Biological Observations of Intervention Group Compared with Placebo Group	Reference; Trial ID
*Gastrointestinal Conditions*					
Regularity of bowel movements in elderly nursing home residents	209; Elderly	*B. longum* 46, *B. longum* 2c	1 x 10^9;^ daily; 7 months	Significantly increased bowel movements without causing diarrhoea	Pitkala et al.^230^
Defecation frequency in elderly patients receiving enteral feeding	Trial 1–83; Elderly Trial 2–123; Elderly	*B. longum* BB536	T1: 5 x 10^10;^ daily; 16 weeks T2: 5 x 10^10^ or 2.5 x 10^10;^ 16 weeks	Significantly normalised defecation frequency in subjects with low & high frequency defecation	Kondo et al.^231^
***Immunity***					
Faecal microbiota & Immune parameters	55; > 84.3 ± 0.98 y	*B. longum* 46, *B. longum* 2c	1 x 10^9^, daily; 7 months	Increased *B. adolescentis* & *B. catenulatum* No significant impact on immune parameters	Ouwehand et al.^232^
Faecal microbiota & Immune parameters	45; ≥ 65 y receiving enteral tube feeding	*B. longum* BB536	5 x 10^10;^ twice daily, 12 weeks;	Numerical increased IgA Maintained NK cell activity	Akatsu et al.^233^
Faecal microbiota & function, & Immune parameters	43; 65–90 y	*B. longum* Synergy 1 prebiotic (FOS & inulin)	2 x 10^11^, 6 g; twice daily, 4 weeks;	Beneficially modulated the gut microbiota; Increased butyrate production; Significantly reduced TNF-α	Macfarlane et al.^234^ NCT01226212
Faecal microbiota & Immune parameters	32; 70 ± 1 y	*B. longum* MM2, *B. bifidum* G9–1, *L. gasseri* KS-13	3 x 10^9^, daily; 3 weeks	Maintained CD4+ lymphocytes; Significantly increased IL-10; Significantly increased bifidobacteria, lactic acid bacteria, decreased *E. coli*	Spaiser et al.^235^ NCT01662206
Physiological status & Immune parameters	98; 84 ± 7.8 y	*B. longum* Bar33, *L. helveticus* Bar13	1 x 10^9^, daily; 30 d	Significantly increased naïve, activated memory, regulatory T cells, B cells, & NK cells & decreased memory T cells	Finamore et al.^236^
***Mood and Cognition***					
Healthy elderly/Cognitive function, Body composition, & Bowel habits	38; 66–78 y	*B. longum* ssp. *longum* BB536, *B. longum* ssp. *infantis M*-63, *B. breve M*-16V *B. breve* B-3 & resistance training	1.25 x 10^10^ 1.25 x 10^10^ 1.25 x 10^10^ 1.25 x 10^10^ daily; 12 weeks	Improved cognitive functioning; Significantly decreased depression-anxiety scores; Significantly improved body mass index scores; Significantly increased frequency of defecation	Inoue et al.^237^ UMIN000021749
Healthy elderly/Cognition, Mood, & Intestinal health	63; ≥ 65 y	*B. longum* BORI, *B. bifidum* BGN4	1 x 10^9^ daily; 12 weeks	Significantly reduced inflammation-associated gut bacteria; Significantly increased serum levels of BDNF; Significantly improved mental attention & executive function; Significantly reduced stress	Kim et al.^238^ KCT0003929

### Mood and cognition

Cognitive aging in older adults has been described as following three different developmental patterns: successful aging, normal aging, and cognitive aging.^246^ During successful aging, cognitive function remains relatively stable. Normal aging is associated with a slight decline in cognitive functioning, while cognitive aging is defined by a steady decline of cognitive ability. Subjective cognitive decline, described as a “self-experienced decline in cognitive ability” has been proposed as the first notable indicator of preclinical Alzheimer’s disease.^247^ In a recent epidemiological study involving 16 cohorts of people from 15 countries (39,387 cognitively unimpaired individuals >60 y of age), subjective cognitive decline was estimated to affect approximately a quarter of the individuals.^248^ Thus, strategies that aid in the management of cognitive decline in elderly subjects could play a significant role in improving quality of life and reducing the risks for dementia and Alzheimer’s disease.

In healthy elderly subjects, a *Bifidobacterium* formulation, containing two *B. longum* strains (*B*. *longum* ssp. *longum* BB536, *B. longum* ssp. *infantis* M-63) and two *B. breve* strains (*B.* *breve* M-16 V, *B. breve* B-3) combined with moderate resistance training improved cognitive functioning as measured via the Japanese version of the Montreal Cognitive assessment instrument.^237^ The strains were associated with a significant decrease in depression-anxiety scores compared with the placebo that included resistance training (*p* = 0.012), based on self-reporting questionnaires. The strains were also associated with improved body mass index scores (*p* < 0.001) and increased frequency of defecation (*p* = 0.023). However, the authors state that the 12-week intervention period was relatively short and represents a limitation of this study. In addition, the sample size of n = 38 limited the statistical power.

In the first well-controlled, multicentre RDBPCT, Kim et al.^238^ investigated the impact of the strains *B. longum* BORI and *B. bifidum* BGN4 on healthy, community-dwelling older adults in terms of cognition, mood, and intestinal health. Twelve weeks of supplementation significantly reduced the abundance of inflammation-associated bacteria including *Eubacterium*, Clostridiales and *Prevotellaceae*. The genus *Allisonella* was also significantly reduced, which is known to produce the biogenic amine histamine that can provoke inflammation.^249^ The formulation also significantly increased serum levels of BDNF compared with the placebo (*p* < 0.05) and this was negatively correlated with strain-associated shifts in *Eubacterium* and Clostridiales. Furthermore, the experimental group exhibited significantly improved mental attention and executive function (*p* < 0.05) and reduced stress (*p* < 0.05). However, the 12-week intervention period is potentially too short – a longer period may reveal changes in some of the cognitive functions for which no significant improvements were noted. The study also lacks direct evidence of improvement in peripheral and cerebral inflammation following consumption of the formulation, thus, further mechanistic studies are required. While neuropsychological assessments were performed by a professionally trained panel, self-reporting was used by participants to assess mood status, which carries the risk of recall bias. Thus, further studies are required to confirm these results. For further details of these trials, see Table 3.

## Conclusion

**Figure 1.**
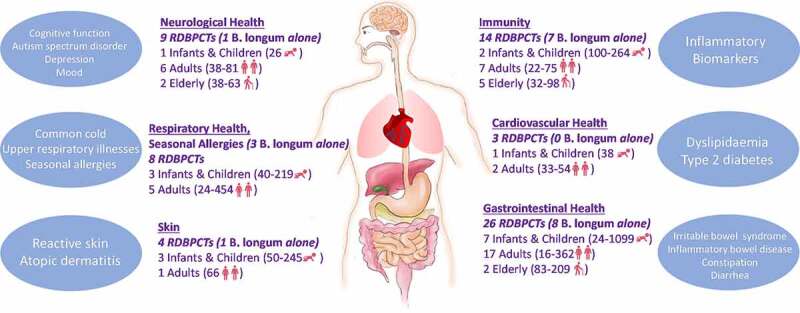
Overview of 64 clinical trials conducted with *B. longum* alone or with other species, and prebiotics. Trials have been categorised according to region/organ of body under investigation. Total number of trials conducted in each category is provided, as well as number of trials conducted in infants and children, adults, and the elderly. The lowest and highest numbers of participants in these trials are included. The most common trial endpoints are provided for each category.

Sixty-four clinical trials have been included in this review with participants ranging from infants to elderly (Figure 1, Tables 1–3). Twenty trials investigated the efficacy of *B. longum* alone, while the remaining 44 trials investigated *B. longum* with other bacterial strains, and/or prebiotics. Significantly, many of the diseases investigated are classified as NCDs, e.g., CVD, diabetes, IBD, or mental health issues. According to the NCD Alliance, the ‘catastrophic expenses’ due to NCD treatment threaten to push 100 million people into poverty each year^250^. Thus, NCDs are recognized as a significant challenge by the 2030 Agenda for Sustainable Development Goals,^251^ and a commitment has been made to reduce a third of premature mortality from NCDs through prevention and treatment.^251^ The trials presented suggest that *B. longum* administration alone or in combination with other bacterial species and prebiotics may have the potential to reduce severity of or prevent certain diseases, including NCDs, in early life, across adulthood and into old age.

However, the trials presented in this review are not without their limitations and in this respect, confirmatory trials are warranted before results can be extrapolated to the appropriate population groups. Many of the trials used insufficient sample sizes, thus diminishing power to detect clinically/biologically significant effects, or randomization was inadequate with prognostic factors unequally distributed across placebo or intervention groups resulting in the over/under estimation of intervention effects. Several authors reported that longer intervention times may have yielded more clinical/biological effects from intervention, thus adequate consideration should be given to the trial duration. Trials that used self-reporting assessments would also benefit from definitive diagnostic tools. Furthermore, crossover trials should be avoided if possible unless the precise washout time is known. But they can also be limited by the fact that they are often performed over different seasons of the year, which can impact host immunity or depression, for example. Thus, these limitations should be avoided. In the future, greater standardization across clinical trials performed with *B. longum* strains alone and in formulations could help provide stronger rationale for more widespread use in the treatment and prevention of disease. This will require greater collaboration between research groups, design of gold-standard, standardized clinical studies that address the same endpoints and biomarkers of health and disease with a focus on dosage and duration, notwithstanding standardized procedures for measuring gut microbiota and metabolome changes to help identify causal mechanisms. However, the evidence to date suggests that continued research and investment into the beneficial properties of *B. longum* is worthwhile given that this species could serve to significantly improve several aspects of human health from birth and beyond.
